# Just Add Data: automated predictive modeling for knowledge discovery and feature selection

**DOI:** 10.1038/s41698-022-00274-8

**Published:** 2022-06-16

**Authors:** Ioannis Tsamardinos, Paulos Charonyktakis, Georgios Papoutsoglou, Giorgos Borboudakis, Kleanthi Lakiotaki, Jean Claude Zenklusen, Hartmut Juhl, Ekaterini Chatzaki, Vincenzo Lagani

**Affiliations:** 1 JADBio Gnosis DA S.A., Science and Technology Park of Crete, GR-70013 Heraklion, Greece; 2 Department of Computer Science, University of Crete, Heraklion, Greece; 3 Institute of Applied and Computational Mathematics, Foundation for Research and Technology, Hellas, N. Plastira 100, Vassilika Vouton, Heraklion, GR-70013 Greece; 4 National Cancer Institute, National Institutes of Health, Bethesda, MD USA; 5 Chief Executive Officer, Indivumed Group, Hamburg, Germany; 6 Laboratory of Pharmacology, Medical School, Democritus University of Thrace, Alexandroupolis, Greece; 7 Institute of Agri-food and Life Sciences, Hellenic Mediterranean University Research Centre, Crete, Greece; 8 Institute of Chemical Biology, Ilia State University, Tbilisi, Georgia

**Keywords:** High-throughput screening, Predictive markers

## Abstract

Fully automated machine learning (AutoML) for predictive modeling is becoming a reality, giving rise to a whole new field. We present the basic ideas and principles of Just Add Data Bio (JADBio), an AutoML platform applicable to the low-sample, high-dimensional omics data that arise in translational medicine and bioinformatics applications. In addition to predictive and diagnostic models ready for clinical use, JADBio focuses on knowledge discovery by performing feature selection and identifying the corresponding biosignatures, i.e., minimal-size subsets of biomarkers that are jointly predictive of the outcome or phenotype of interest. It also returns a palette of useful information for interpretation, clinical use of the models, and decision making. JADBio is qualitatively and quantitatively compared against Hyper-Parameter Optimization Machine Learning libraries. Results show that in typical omics dataset analysis, JADBio manages to identify signatures comprising of just a handful of features while maintaining competitive predictive performance and accurate out-of-sample performance estimation.

## Introduction

The number of molecular biological datasets available (defined as collections of molecular profiles of several biological samples) is increasing at a rapid pace, presenting welcoming opportunities for new science. Public repositories such as Gene Expression Omnibus^1^, recount2^2^, Metabolomics Workbench^3^, and the NCI Genomics Data Commons^4^ collectively count hundreds of thousands of datasets. The samples in those datasets are typically associated with an outcome (target, dependent variable) of interest, such as disease status, response to treatment, disease sub-type, quantitative phenotypic trait, and time to an event of interest (e.g., survival, complication, metastasis). Predictive model instances (hereafter, models) can be learned (fit, constructed) to predict the outcome in future unseen (out-of-sample) profiles, using modern statistical and machine learning methods. This type of analysis is known in the machine learning field as supervised learning^5^, as opposed to unsupervised (clustering)^5^ and self-supervised^6^ learning approaches used for unlabeled samples. i.e., when there is no specific outcome of interest to predict. In supervised learning, methods falling in the class of feature selection (a.k.a., variable selection or attribute selection), can be coupled with predictive modeling algorithms to identify (bio)signatures, defined as minimal-size subsets of molecular and other biological measurements that collectively lead to optimal predictions. Identifying such predictive signatures is of major importance for knowledge discovery, gaining intuition into molecular pathophysiological mechanisms, identifying novel drug targets, or designing diagnostic/prognostic assays with minimal measuring requirements. Feature selection for knowledge discovery is often the primary goal in an analysis and the predictive model is just a side-benefit. Feature selection is different than differential expression analysis: feature selection examines feature correlations in combination (multivariately) and not individually. Not only it removes irrelevant features, but also features that are redundant for prediction given the selected ones.

Despite the plethora of available data, algorithms, and computational power, a major bottleneck in molecular data analysis is still present: lack of human experts’ time. In addition, analyses are prone to methodological errors that invalidate results and mislead the community^7^. Is it possible to fully automate the sophisticated, advanced multivariate analysis of biological data and the discovery of biosignatures? Can we democratize machine learning to life scientists and non-expert analysts? Can we reduce statistical methodological errors that creep into analyses? Quite importantly, can we identify small size signatures that carry all predictive information of the outcome in typical omics analyses?

As a response to the above challenges, the field of Automated Machine Learning (AutoML) recently emerged^8^. AutoML tools try to automate the analysis and predictive modeling process end-to-end in ways that provide unique opportunities for improving healthcare^9^. They automatically try thousands of combinations of algorithms and their hyper-parameter values to obtain the best-possible model. However, state-of-the-art AutoML tools do not cover all the analyses needs of the translational researcher. Firstly, they do not focus on feature selection. As a rule, they return models that employ and require to measure all molecular quantities in the training data to provide predictions, thus hindering interpretation, knowledge discovery, and the ability to translate to cost-effective benchtop assays. Secondly, they do not focus on providing reliable estimates of the out-of-sample predictive performance (hereafter, performance) of the models. The latter is particularly important to a practitioner gauging the clinical utility of the model. Thirdly, they do not provide all information necessary to interpret, explain, make decisions, and apply the model in the clinic.

Focusing on reliable performance estimation, we note that it is particularly challenging in omics analyses for at least three reasons. The first one is the low-sample size of typical omics datasets^10^. It is not uncommon for biological datasets to contain fewer than 100 samples: as of October 2021, 74.6% of the 4348 curated datasets provided by Gene Expression Omnibus count 20 or fewer samples. This is typical in rare cancers and diseases, in experimental therapy treatments, and whenever the measurement costs are high. When sample size is low, one cannot afford to hold out a significant portion of the molecular profiles for statistical validation of the model. The second reason is that trying multiple machine learning algorithms or pipelines to produce various models and selecting the best-performing one, leads to systematically overestimating its predictive performance (bias). This phenomenon is called the “winner’s curse” in statistics^11^ and the multiple comparisons in induction algorithms problem in machine learning^12^. The third reason is that omics datasets measure up to hundreds of thousands of features (dimensions). Such high dimensional data are produced by modern biotechnologies for genomic, transcriptomic, metabolomic, proteomic, copy number variation, single nucleotide polymorphism (SNP) GWAS profiling, and multi-omics datasets that comprise of multiple modalities. The combination of a high number of features (*p)* and a low-sample size *(n*), or as it is called “large *p*, small *n*” setting, is notoriously challenging as it has been repeatedly noted in statistics^13^ and bioinformatics^14,15^ leading to problems of model overfitting and performance overestimation. Such challenges have been recently noted in the precision medicine research community as well^10,16^.

In this work, we describe the design and properties of a web based AutoML platform to address the above challenges, called Just Add Data Bio or simply JADBio, version 1.2.24 (April, 1, 2021). JADBio is qualitatively compared against auto-sklearn^17^, TPOT^18,19^, GAMA^20^, AutoPrognosis^21^, and Random Forests^22^, demonstrating that it provides a wealth of unique functionalities necessary to a translational researcher for decision support and clinically applying a model. A case-study on the microbiome of colorectal cancer is presented to illustrate and demonstrate JADBio’s functionalities. JADBio is also comparatively and quantitatively evaluated on 360 public biological datasets, spanning 122 diseases and corresponding controls, from psoriasis to cancer, measuring metabolomics, transcriptomics (microarray and RNA-seq), and methylomics. It is shown that, on typical omics datasets, JADBio identifies signatures with just a handful of molecular quantities, while maintaining competitive predictive performance. At the same time, it reliably estimates the performance of the models from the training data alone, without losing samples to validation. In contrast, several common AutoML packages are shown to severely overestimate the performance of their models.

## Results

### Case-study problem definition: analyzing colorectal cancer microbiome data

We present a case-study example to showcase the functionalities of JADBio for predictive modeling, biosignature discovery, and its pertinence to oncology and molecular data analysis in general. We use data from Wirbel et al.^23^, where five public cohorts of colorectal cancer cases and controls and their microbial gut profiles were assembled. The profiles were uniformly preprocessed to allow pooling them together. The cohorts were named after the corresponding country where the sampling was performed (China CN^24^, France FR^25^, United States US^26^, Germany DE^23^, and Austria AT^27^). Collectively, the five cohorts contain 575 samples (285 colorectal cancer cases, 290 healthy controls). The samples were profiled with shotgun metagenomics measuring the relative abundance of 849 microbial species. We used these data to replicate part of the analyses presented in Wirbel et al.^23^. The analysis tasks are: (a) to create a predictive, diagnostic model for colorectal cancer given a microbial gut profile, (b) to estimate the predictive performance of the model, (c) to identify the biosignature(s) of microbial species that predict colorectal cancer, and (d) to apply the colorectal cancer model on new data. Particularly, we use each cohort to build a predictive model and every other cohort as its external validation set. The detailed set of results is presented in Supplementary Table 1. Overall, JADBio analyses deliver similar performances as in the original publication^23^, with the maximum difference between original and JADBio analyses being 0.08 AUC points. However, note that JADBio requires minimal human effort and selected fewer predictive features on average (up to 25). The number of features used in Wirbel et al.^23^ for each model cannot be extracted as they use an ensemble of models’ approach; for all practical purposes their models employ the full set of 849 features. Using all measured biomarkers in the model limits the knowledge discovery aspect of their analysis and their application to designing diagnostic assays.

### Case-study: create a diagnostic model instance for colorectal cancer given a microbial gut profile

To initiate an analysis with JADBio using the GUI, the 2-dimensional training data matrix with the measurements is uploaded, the user designates which column contains the outcome, and the analysis begins; during the analysis, the user can monitor its progress (see Supplementary Fig. 1 for details). All GUI functionalities, as well as additional ones, are also available through a REST API interface or a Python API wrapper (see Software Availability). In the case of the CN cohort, the analysis completes within 44 min after training 127160 model instances stemming from 3179 configurations (i.e., machine learning pipelines each of which combines different algorithms with different tuning hyper-parameters; see Methods. A configuration accepts the data and produces a predictive model). The user can share results with the community for reproducibility purposes and interactive examination through unique sharable links. Results from the CN cohort are available at this link: https://app.jadbio.com/share/f138ced3-1357-465b-81f0-523e64a3abf7. The expert analyst can look at the analysis report with a description of all computations that took place, including all configurations that were tested (https://app.jadbio.com/share/f138ced3-1357-465b-81f0-523e64a3abf7/report). Links for the analyses of the other cohorts are available in Supplementary Table 2. The results and analysis performed are summarized in an automatically generated natural language report for inclusion in scientific publications (an example of such reports is presented in the companion webpage, https://jadbio.com/jadbio-extensive-evaluation-resource-page/).

JADBio reports configurations optimized for different criteria, namely performance, interpretability, feature selection, and aggressive feature selection. When the Performance criterion is selected, JADBio reports the configuration with the highest expected predictive performance. When Interpretability is selected, JADBio reports the best-performing configuration among those whose predictive algorithm generates models that are humanly interpretable. When either Feature Selection or Aggressive Feature Selection is selected, JADBio reports the best configuration when Feature Selection is integrated in the pipeline, with Aggressive Feature Selection enforcing the identification of minimal size feature subsets at the expense of reduced performance, on average.

**Fig. 1:**
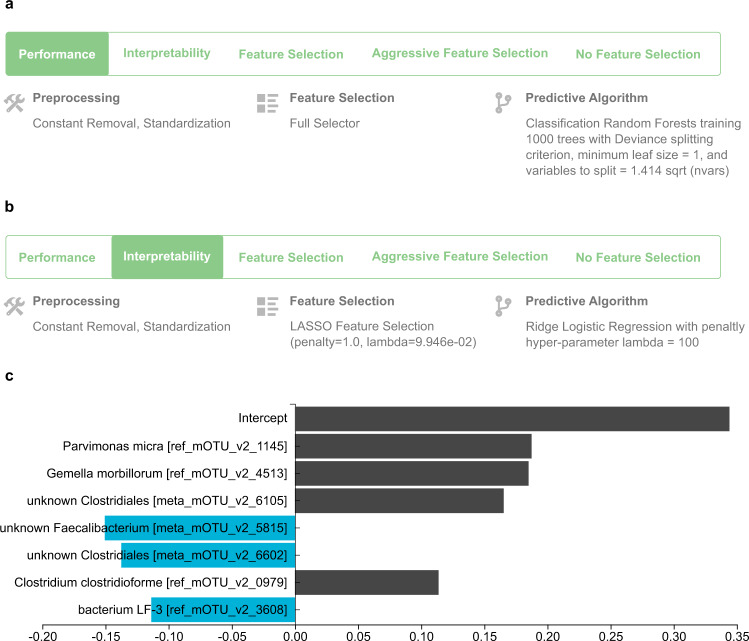
The best performing and best interpretable model trained on the China (CN) cohort. **a** Winning configuration that leads to the production of the final best-performing model when applied to all training data. It is produced by first applying removal of constant-value features and standardization of all features, followed by including all features in the model (FullSelector, i.e., no feature selection), and then modeling using the Random Forest algorithm. The hyper-parameter values to use are also shown. **b** Configuration that leads to the production of the best interpretable model. It is a linear Ridge Logistic Regression type of model, preceded by a Lasso regression for feature selection. **c** The interpretable model can be visualized, in contrast to the best-performing one. JADBio shows the standardized coefficients of the model for each selected feature and the intercept term.

The configuration that produces the best-performing model on the CN cohort is reported in Fig. 1a. It produces a non-linear model, specifically, a Random Forest that includes 1000 different decision trees. If “Interpretability” is selected as criterion for this analysis, the best interpretable model is a linear Ridge Regression type of model (Fig. 1b), whose standardized coefficients are shown in Fig. 1c.

### Case-study: estimating the predictive performance of the colorectal cancer model

The predictive model outputs the probability of a sample to belong to a given class; in this case, it is the probability of having colorectal cancer. The user can decide on a clinically relevant classification threshold *t* and call as cancerous any sample with predicted probability higher than *t*. Several metrics of performance depend on this threshold *t*, such as sensitivity, specificity, accuracy, precision, and positive predictive value.

**Fig. 2:**
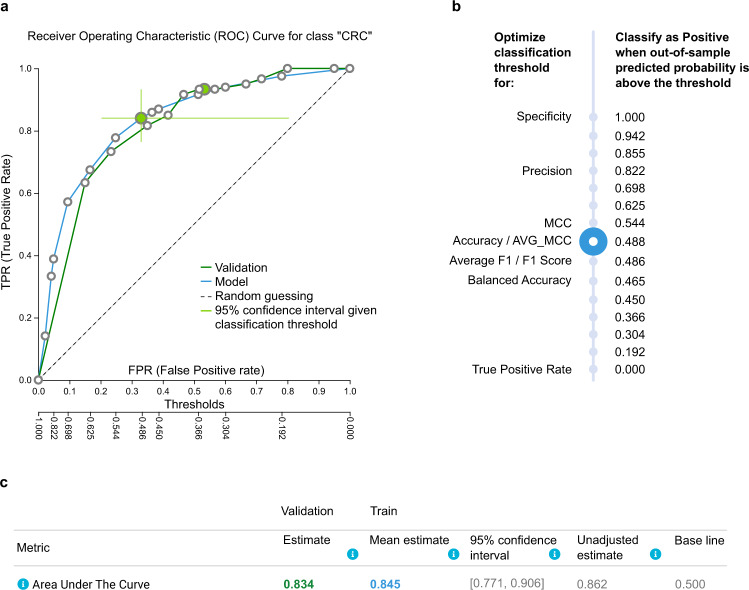
Predictive performance of the winning model optimized for Feature selection, trained on the China (CN) cohort and validated on the Germany (DE) cohort. **a** The Receiver Operating Characteristic (ROC) curve estimated on the training set and controlled for trying multiple different configurations is shown as the blue line. It shows all trade-offs between False Positive Rate (top *x*-axis) and True Positive Rate (*y*-axis) for all different classification thresholds (bottom *x*-axis). By clicking on a circle, a corresponding threshold is selected. The user can see how metrics of performance are affected, which facilitates selection of the clinically optimal threshold. The green cross shows the confidence intervals in each dimension for that point on the ROC. The ROC curve achieved by the model’s predictions on the DE cohort used as external validation set is shown as the green line. It closely follows the blue line estimated from the training. **b** A list of different thresholds suggested by JADBio and the corresponding metric of performance that they optimize. E.g., accuracy is optimized for threshold 0.488, while balanced accuracy is optimized for 0.465. **c** The threshold-independent metric ROC Area Under the Curve (AUC) as estimated by the training (bold blue) and achieved on the validation (bold green) is reported along with its confidence intervals. Its unadjusted estimate (i.e., not adjusted for trying multiple configurations) is also reported and is expected to be optimistic on average. All other estimates computed by JADBio, including but not limited to accuracy, precision, recall (not shown in the figure) are adjusted for trying multiple configurations as well.

Figure 2 reports some of the information JADBio provides on predictive performances for the model trained on the CN cohort and optimized for “Feature Selection”. Specifically, it presents the expected, out-of-sample Receiver Operating Characteristic curve (ROC) that shows all trade-offs between the False Positive Rate and True Positive Rate achieved by using different thresholds on the output of the final model (Fig. 2a, blue line). By clicking on a point on the ROC or the skyscraper in Fig. 2b, the clinician can select a different threshold leading to different sensitivity, specificity, or accuracy. For example, the blue circle selected in Fig. 2b corresponds to a probability classification threshold of 0.488, which leads to 0.841 True Positive Rate (TPR), and 0.33 False Positive Rate (FPR). Using this threshold, the model operates on the green circle on the ROC of Fig. 2a. The green cross on Fig. 2a displays the 95% confidence interval in terms of both TRP and FPR. In a similar fashion, the Precision-Recall curve shows all achievable trade-offs between precision and recall (Supplementary Fig. 2). If the given FPR is not acceptable for the clinical application of the model, another circle/threshold could be selected.

The Area Under the ROC Curve (AUC) is a threshold-independent metric of performance, as its computation sums over all possible threshold values. For the model in Fig. 2a, the AUC turns out to be 0.845; confidence intervals and the unadjusted AUC value are reported as well. The latter is the estimate without adjusting (controlling) for trying multiple configurations and, as shown empirically and theoretically in prior work^28^, systematically overestimates the performance on unseen profiles. Except for metrics specifically denoted as “unadjusted”, we would like to note that all estimates and confidence intervals reported by JADBio are adjusted for trying numerous configurations (winner’s curse) and are, in general, conservative (see Methods); this includes not only the AUC but all metrics for all thresholds displayed (see Supplementary Fig. 2). This means that the user can optimize the classification threshold for clinical use to the best sensitivity/specificity trade-off without overfitting.

As an example of an external validation of a model, we apply the Feature Selection CN model on the DE cohort. The AUC achieved on the validation set is 0.834 (Fig. 2c) close to the estimated value of 0.845 from the training set and within the confidence interval. The unadjusted estimate (0.862) seems on the overoptimistic side, but still within the confidence intervals in this case. The ROC curve achieved on the validation set is shown as the bold green line in Fig. 2a.

### Case-study: identify the biosignature(s) of microbial species that predict colorectal cancer

**Fig. 3:**
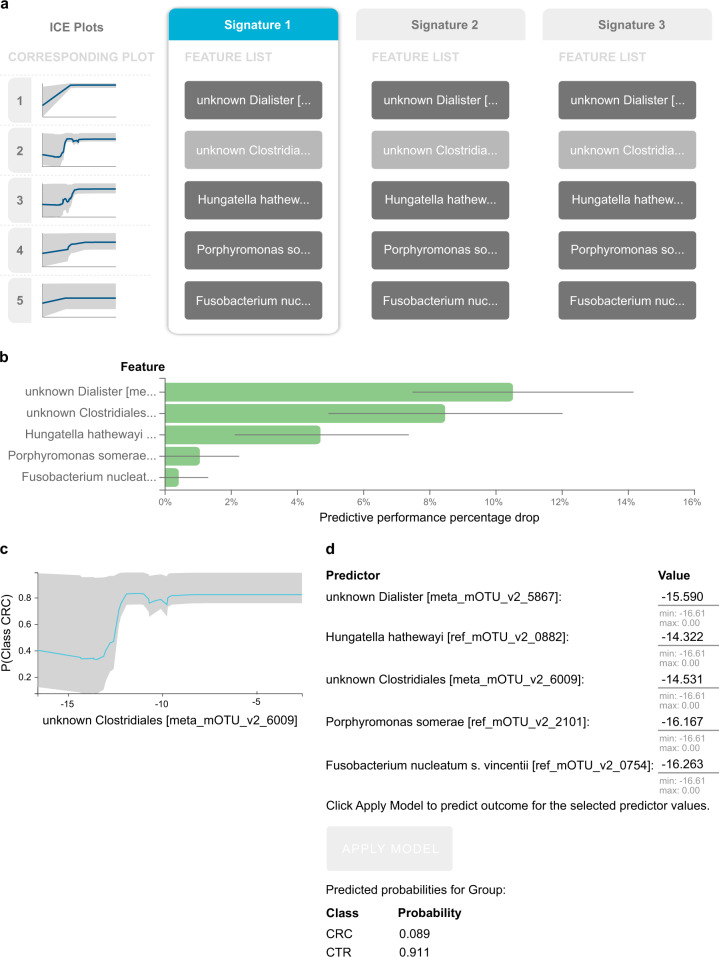
Feature selection (biosignature discovery) results for the best-performing model using the France (FR) cohort as training data. **a** There are three biosignatures identified, each containing five features. Each signature leads to an optimally predictive model. They share four features, while the second feature varies across signatures. **b** Feature importance plot, assessing the relative drop in performance if each feature is dropped from the signature and the rest are retained. **c** The Individual Conditional Expectation (ICE) plot for the ‘unknown Clostridiales [meta_mOTU_v2_6009]’: the higher its abundance, the higher the probability of colorectal cancer. Note that the trend is non-linear and non-monotonic (close to step function). **d** The manual application of the model to get predictions for a fictional new sample is shown. Values are log-transformed relative concentrations, which can be negative if the original numbers are smaller than the logarithm base.

JADBio performs feature selection (biosignature discovery) simultaneously with modeling to facilitate knowledge discovery. JADBio performs multiple feature selection, meaning that it may identify multiple alternative feature subsets that lead to equally predictive models, up to statistical equivalence, if present. Among the performed analyses, multiple signatures were identified only in the FR cohort. The best-performing model for this cohort presents three biosignatures (Fig. 3a), each containing just five features (i.e., microbe species) selected out of a total of 849 features. The first feature for all signatures, named ‘unknown Dialister’, is the relative abundance of an unknown microbe from the Dialister genus. The second feature varies across the signatures among three unidentified, yet possibly distinct microbes from the Clostridiales order, denoted as “unknown Clostridiales [meta_mOTU_v2_6009]”, “unknown Clostridiales [meta_mOTU_v2_5514]”, “unknown Clostridiales [meta_mOTU_v2_7337]” (full names trimmed out in Fig. 3a). In other words, the abundance of each of these three microbes could substitute for the others in the signature and lead to an equally predictive model: they are informationally equivalent with respect to the prediction of this outcome.

Interestingly, while the unknown Dialister and the Hungatella hathewayi microbes respectively rank first and second among the markers most associated (pairwise) to the outcome (two-tailed *t*-test), the Porphyromonas somerae and Fusobacterium nucleatum (univariate, unconditional) *p*-values rank in 27th and 46th positions, respectively. This example anecdotally illustrates the difference between standard differential expression analysis that examines genes independently, and feature selection that examines biomarkers jointly^29^. Features with relatively weak pairwise association with the outcome may be selected in a multivariable analysis because they complement each other towards predicting the outcome.

JADBio presents Individual Conditional Expectation (ICE) plots (see Methods and Goldstein et al., 2015^30^) to facilitate the interpretation of the role of the features in the model. Figure 3c presents the ICE plot for one of the unknown Clostridiales microbe. The *y*-axis corresponds to the predicted probability that the sample belongs to a subject diagnosed with colorectal cancer. The bold blue line in the plot shows how the average of the predicted probability changes against the value of the marker. The gray area indicates the range of the predictions due to the values of the other biomarkers. The higher the abundance of the specific microbe, the higher the probability of disease on average; hence, we would call Clostridiales overabundance a risk factor for the disease. The Feature Importance graph (Fig. 3b) shows the relative drop of predictive performance when a single feature is removed from the model. In this case, removing the Dialister feature from the model is expected to cause a relative drop in performance slightly above 10%, while removing the second feature would have a smaller effect (around 8%).

### Case-study: apply the colorectal cancer model to new, unseen data

The users can apply the model in batch form on a new labeled dataset for external validation, or on an unlabeled dataset to get predictions, or even download the model as executable to embed it in their own code. They can also manually input the values of the predictors and get a single prediction. This is a useful functionality to explore what-if scenarios of how observed feature values affect predictions (we warn however, against interpreting causally these scenarios, i.e., when feature values are not observed but manipulated). As an example, the manual application of the best-performing model for the FR cohort is shown in Fig. 3d.

Overall, JADBio managed to create accurate diagnostic models for colorectal cancer from microbial gut data that transfer across different populations. JADBio identified signatures of up to 25 features, out of the 849 available ones. Model construction did not require any coding.

### Qualitative comparison against state-of-the-art AutoML tools

We evaluate and qualitatively compare several AutoML platforms in terms of provided functionalities, namely auto-sklearn^17^, TPOT^18^, TPOT-MDR^19^, GAMA^20^, and AutoPrognosis (AP^21^). The functionalities indicate the scope of applicability of the platform, as well as the breadth of covering the needs of a translational researcher. In Table 1, the presence of a functionality is indicated with a ✓ symbol. Asterisks are explained in the text below.

- CASH: Combined Algorithm Selection and Hyperparameter optimization is the ability to automatically try numerous combinations of algorithms and their hyper-parameter values to identify the one(s) to produce a final predictive model. CASH methodology directly affects the quality of the models produced.
- (Multiple) Biosignature Identification (Feature Selection): Ability to select feature subsets that are of minimal size and optimally predictive. This removes irrelevant but also redundant features. It is the main tool for knowledge discovery. Returning multiple such subsets provides choices to designers of clinical multiplex assays (see also Methods). TPOT-MDR does not guarantee biosignature identification, but it does occur occasionally (denoted by an asterisk in the corresponding cell).
- Feature Interpretation plots, like the ICE plot^30^ help the biomedical scientist interpret the role of a biomarker as a risk factor, a protective factor, or something more complex (risk or protection depends on context). The “Selected Feature Added Value” indicates the effect of the removal of this feature from the model. It helps the designer of laboratory assays of clinical value to gauge the cost effectiveness of including the marker in the assay.
- Predictive Performance Estimation is necessary to judge the quality of a model before clinical application. JADBio embodies a combination of two algorithms in this category (see Methods for more details). The first is the Generalized cross validation (GCV) algorithm that estimates the out-of-sample performance of each configuration to select the winning one. GCV is a generalization of the standard CV that can vary depending on the sample size and imbalance of the outcome classes. The second one is the Bootstrap Bias Correction Cross Validation (BBC-CV) protocol to correct the estimate of the winning configuration for the “winner’s curse”. The other tools apply CV for each configuration which is expected to overestimate performance systematically. As a result, it is necessary to withhold a separate test set and lose samples to estimation. The asterisk on auto-sklearn and GAMA indicates that performance estimates are not provided in the user output but need to be extracted from their log files. Confidence Intervals help the clinician gauge the uncertainty of the quality of the model to interpret its practical value.
- Clustered or grouped samples (not to be confused with clustering of samples) are profiles that are sampled in a correlated way, e.g., repeated measurements on the same cell culture or human subject. Ignoring the grouping of samples may lead to overestimated performances.
- Optimization of clinical thresholds: JADBio facilitates the choice of the classification threshold for optimal clinical use to achieve the best trade-off between sensitivity and specificity (see Methods for more details).

**Table 1:** AutoML tools functionalities. The presence of a functionality in each tool is marked by a check symbol or by a short name/acronym. Asterisks and short names are explained in the text.

	JADBio	auto-sklearn	TPOT	TPOT-MDR	GAMA	AP
CASH^*^	✓	✓	✓	✓	✓	✓
BioSignature Identification^*^	✓			✓^*^		
(Multiple) Signatures Identification	✓					
Selected Feature Interpretations	ICE plot					
Selected Feature Added Value	✓					
Explains individual predictions						✓
Predictive Performance Estimation	GCV, BBC-CV	Holdout, CV^*^	CV	CV	CV^*^	CV
Confidence Intervals	✓					✓
Classification (nominal) outcome	✓	✓	✓	✓	✓	✓
Regression (continuous) outcome	✓	✓	✓	✓	✓	
Time-to-event (survival) outcome	✓					
Optimization of Clinical Thresholds	✓					
Accepts missing values	✓	✓	✓	✓	✓	✓
Handles clustered samples	✓	✓				

### Quantitative study: large-scale, comparative evaluation on public datasets set up

Computational experiments were conducted on a large corpus of 360 high-dimensional datasets, including transcriptomics (271 microarray, 23 RNA-seq), epigenomics (23), and metabolomics (43) data. The datasets are related to 125 diseases or phenotypes, with cellular proliferation diseases (i.e., different types of cancers) being the most represented group with 154 datasets. Supplementary Data 1 lists all datasets and respective characteristics. We run JADBio by optimizing four different criteria, namely Performance (JADBio-P), Interpretability (JADBio-I), Feature Selection (JADBio-FS), and Aggressive Feature Selection (JADBio-AFS). We compare against auto-sklearn^17^, TPOT^18^, GAMA^20^, AutoPrognosis^21^, and Random Forest (RF^22^, scikit-learn implementation^31^). Each dataset was partitioned into two equal-size sample sets, each serving as the train and test set, respectively, leading to a total of 720 analyses. Further details in the Methods.

### Quantitative study: JADBio enables knowledge discovery and provides highly predictive models

**Fig. 4:**
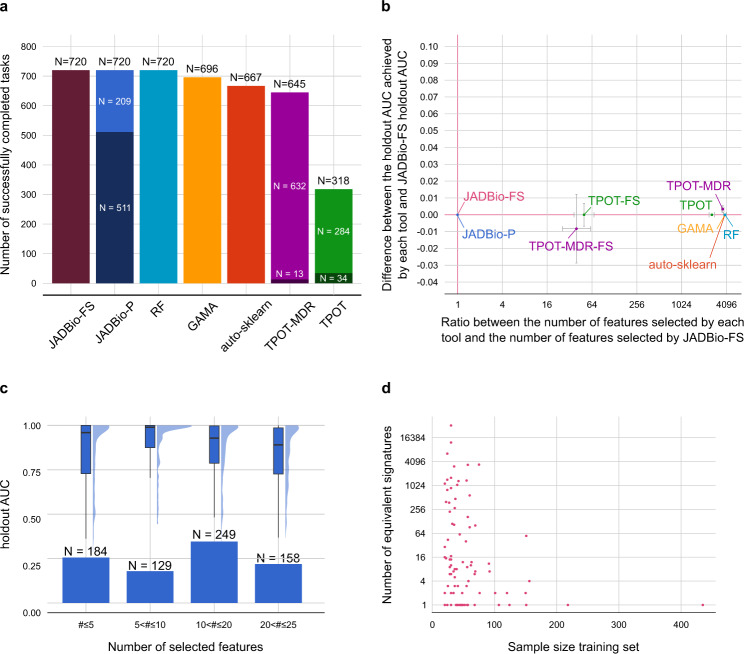
Results of the quantitative comparison in terms of predictive performance. **a** Number of successfully completed tasks out of 720 for each tool. AutoPrognosis never completes any run and is not shown. JADBio-P and TPOT-MDR do attempt feature selection. The portion of runs where feature selection led to a winning model is depicted with a darker shade in their respective columns. JADBio-FS always enforces feature selection. **b** Comparison between JADBio-FS and all other tools in terms of relative dimensionality reduction and performance gain. The dimensionality reduction with respect to JADBio-FS (*x*-axis) is computed as the ratio between the number of features selected by each tool and the number of features selected by JADBio-FS (log2 scale). Values larger than one indicate that the tool selects more features than JADBio. The performance gain is reported on the *y*-axis, and it is computed as the difference between the holdout AUC achieved by each tool and the holdout AUC achieved by JADBio-FS. Values larger than zero indicate that the tool performs better than JADBio-FS. For each tool, we plot a point on the median values for each axis. Errors bars showing the standard error are shown. The median values are computed only on the completed runs. Cranberry-colored lines denote the baseline value, i.e., relative dimensionality reduction equal to one and performance gain equal to zero. **c** Holdout AUC distribution for JADBio-FS over different number of selected features. The holdout AUC distribution is reported both as a box and violin plot. **d** Contrasting the sample size in the training set (*x*-axis) and the number of equivalent signatures identified by JADBio-FS (*y*-axis, log2 transformed). Only runs where an algorithm able to identify multiple signatures was selected are shown. More than one signature was identified in a total of 68 runs out of 98, represented by the points with y coordinate larger than 1.

Figure 4 reports the quantitative evaluation results. JADBio (all settings) and RF successfully completed all 720 analyses (Fig. 4a, Supplementary Fig. 3, Supplementary Table 3). GAMA and auto-sklearn completed 696 (96.7%) and 667 (92.6%), respectively, while TPOT-MDR and TPOT completed 645 (89.6%) and 318 (44.2%) runs. Failed runs either reached the time limit or were interrupted by internal errors, with the latter being the most common reason (Supplementary Table 3). AutoPrognosis failed to complete any analysis and is omitted from the figures. Specifically, in 405 (56.3%) datasets runs were halted because at least 50 samples were required to proceed with an analysis.

Figure 4b reports the median performance difference between a given tool and JADBio-FS (*y*-axis, smaller is better for JADBio-FS) against the median ratio of the number of selected features by a given tool and JADBio-FS (*x*-axis, smaller is better). Medians are computed only on the runs completed successfully. All tools are on par with JADBio-FS with respect to performance, except TPOT-MDR which shows a small (0.0034 AUC points), yet statistically significant advantage and JADBio-AFS which is statistically significantly outperformed by JADBio-FS by 0.011 AUC points (Bonferroni-adjusted *p*-values < 0.05, Supplementary Figs. 4 and 5, Supplementary Table 4). When the average difference is considered instead of the median, all tools except JADBio-AFS outperform JADBio-FS’s, however the differences are often negligible. Specifically, the differences are <0.01, <0.01, 0.011, 0.017, and 0.031 against RF, GAMA, TPOT, auto-sklearn, and TPOT-MDR, respectively. Only the differences with TPOT-MDR and auto-sklearn are statistically significant (Bonferroni-adjusted *p*-value < 0.05, Supplementary Figs. 6 and 7, Supplementary Table 5).

The small performance advantage comes at the price of a disproportionately large number of selected features. All tools other than JADBio never or rarely apply feature selection, meaning that their models are based on all available features. The default maximum number of features to select for JADBio-FS is 25, however, in most cases the upper limit is not reached (Fig. 4c). This leads to an average dimensionality reduction of ~4000 times against all other tools. Even when we focus on the 13 and 34 runs where TPOT-MDR and TPOT do employ models with selected features (points labeled as TPOT-MDR-FS and TPOT-FS in Fig. 4b), they select a median of 164 and 197 features respectively, an amount five times larger than what JADBio-FS selects, without any statistically significant difference in performances (median difference: -0.008 and 0.0 for TPOT-MDR and TPOT, respectively).

Figure 4c reports the distribution of the number of selected features binned in four different groups with respect to the number of selected features. The distribution of the holdout AUC for each category is also plotted at the top of the panel. In almost half of the runs (313, 43.5%) JADBio-FS selects fewer than ten features. Median holdout AUC performances for the four categories are 0.96, 0.99, 0.93, and 0.89, sorted from the fewest to more selected features. Models containing 5 < #features ≤ 10 features lead to statistically significantly higher AUC than models with a larger number of selected features (Bonferroni-adjusted Wilcoxon-test *p*-value 0.002 and 1.69e-6, respectively).

In Fig. 4d, we examine the identification of multiple signatures. We focus on the 98 runs where there was a possibility to identify multiple signatures, namely the runs where the winning configuration contained a feature selection algorithm able to identify multiple signatures. JADBio-FS detects multiple signatures in 68 (69.39%) out of these 98 runs. The average number of signatures is equal to 1106, peaking at 33360 (Fig. 4d). The number of equivalent signatures tends to decrease with sample size (not in a strongly statistically significant way, Pearson correlation -0.237, p-value 0.052). This could be explained by the fact that when sample size is low, it is harder to discern the truly optimal signature^32^.

The results show that JADBio leads to models that require about 4000 fewer biomarkers to measure to achieve comparable predictive performance against all other tools included in the experiments.

### Quantitative study: JADBio avoids overestimating performances

**Fig. 5:**
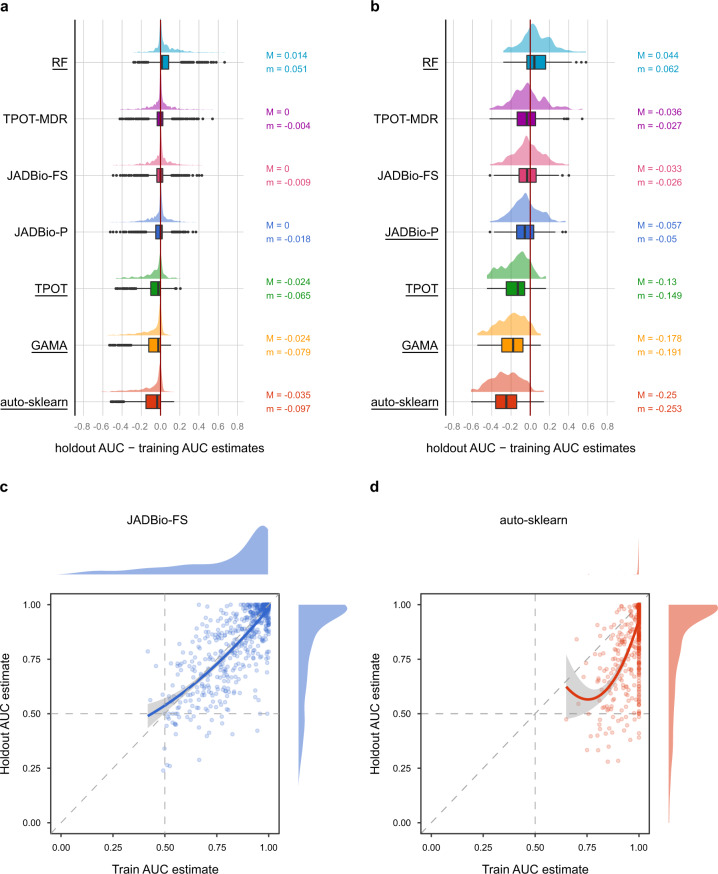
Results of the quantitative comparison in terms of bias. Bias is defined as the holdout AUC estimate minus the AUC estimated on the training set; distributions on the right of the zero line correspond to conservative estimates, while results on the left of the line are optimistic. **a** AUC bias distribution across 720 classification tasks (2 tasks for each of the 360 datasets). The distribution for each tool is reported both as a box and violin plot. Tools with median bias statistically significantly different from zero have their name underscored. Median (M) and average (m) bias values are reported on the right of each boxplot axis. **b** Same as **a**, but only for the 196 runs where Random Forest (RF) achieves less than 0.8 AUC on the holdout set. On these more difficult tasks, TPOT, GAMA, and auto sklearn systematically overestimate performance between 14 and 25 AUC points. **c** and **d** Scatterplots reporting the AUC estimated on the training set (*x*-axis) vs. the AUC estimated on the holdout set (*y*-axis) for the JADBio-FS and auto-sklearn tools, respectively. Each point corresponds to one run. Locally Weighted Scatterplot Smoothing (LOESS) regression lines indicate the main trends in the corresponding scatterplot. Auto-sklearn points and LOESS line are mostly below the diagonal, indicating systematically overoptimistic performance estimates on the training set. The bias is significantly reduced for JADBio-FS, as indicated by a LOESS regression line close to the diagonal.

We investigate whether performance estimates obtained from the training set accurately reflect the ones computed on the holdout set. This is important to correctly assess the clinical usefulness of a model. The estimation bias is defined as the difference between the AUC estimated on the holdout set and the one estimated on the training set. A positive bias indicates systematically conservative estimates, while a negative one indicates optimistic estimates. Figure 5a reports the bias for the five tools with the highest predictive power. JADBio (all settings) uses Bootstrap Bias Corrected (BBC) cross validation (CV), a protocol specifically devised for removing the estimation bias^28^. RF uses Out Of Bag (OOB) estimation^22^, auto-sklearn an internal holdout approach, and all other tools employ the uncorrected cross-validation estimate of the winning configuration^33^. We’d like to note however, that the authors of GAMA and auto-sklearn are aware of the estimation problems and warn against the use of the training estimates without further validation on a separate holdout set.

Figure 5a shows the distribution of the bias, along with the median (M) and average (m) for each tool. JADBio-FS, JADBio-P, and TPOT-MDR median estimation bias is statistically indistinguishable from zero (Fig. 5a, Supplementary Fig. 8, Supplementary Table 6). RF using the OOB^22^ estimation protocol underestimates performance having a strong positive bias (median value 0.014). The tools auto-sklearn, GAMA, TPOT statistically significantly overestimate performance with median bias values of (−0.035), (−0.024), and (−0.024), respectively (Bonferroni-adjusted *p*-value < 0.05, see also Supplementary Table 6). The average bias for auto-sklearn, GAMA, TPOT, and JADBio-P are respectively (−0.1), (−0.08), (−0.065), and (−0.018), respectively (Supplementary Table 7). Average bias values are in general more extreme than the median ones due to the presence of outliers (Fig. 5a).

Although these levels of bias seem relatively innocuous, it is a misleading conclusion due to a ceiling effect: numerous models have high predictive power and hence there is no opportunity to overestimate performance in these runs. We now restrict attention to the more difficult tasks, specifically to the ones where RF achieves an AUC less than 0.8 on the test set. The results are shown in Fig. 5b and Supplementary Table 8. The median bias of auto-sklearn in these runs is −0.25. This means that auto sklearn may estimate the performance of a model to 0.75 AUC from the training set, when the true performance on unseen data is equal to random guessing 0.5 AUC. Similar results on auto-sklearn were reported also in another recent publication^34^.

Panels (c) and (d) of Fig. 5 better visualize the difference in estimation properties between AutoML platforms. Each dot corresponds to a single run, where the *x*-axis reports the AUC estimate on the training set and the *y*-axis the estimate on the holdout set. The estimated training AUC is plotted against the achieved holdout AUC. Points above/below the diagonal correspond to runs underestimating/overestimating performance. The LOESS regression lines are shown in bold. Notice that auto-sklearn almost always overestimates performance. The stripe of dots on the right of the panel corresponds to auto sklearn estimates of exactly AUC = 1, while their holdout AUCs range from less than 0.5 to a perfect score of 1. In contrast, JADBio-FS runs are symmetrically distributed around the diagonal demonstrating that JADBio-FS estimates from the training set are on average accurate (zero bias). Of course, there is still variance in the estimation, which is why JADBio also reports confidence intervals. Other JADBio settings have more conservative performance estimates than JADBio-FS, possibly due to lower numbers of configurations explored (Supplementary Table 9).

In summary, these results indicate that JADBio provides reliable generalization performance estimates from the training set alone, while auto sklearn, GAMA, and TPOT fail in this respect.

## Discussion

JADBio can analyze binary, multi-class (classification), right-censored time-to-event (survival analysis), and quantitative (regression) outcomes. It accepts nominal and continuous predictive features, medical images (e.g., histopathological, x-rays, and cell) and it has been applied to medical signals as well (e.g., electrocardiogram, ballistocardiogram)^35^. The dataset may contain multi-omics measurements, clinical, epidemiological, or lifestyle measurements. However, within the scope of this paper we focus only on binary classification tasks from single omics datasets.

The results support the following claims. First, JADBio provides useful automation and functionality for translational research, predictive modeling, and corresponding decision support. Analyses handled may involve multi-omics data complemented with clinical, epidemiological, and lifestyle factors resulting in hundreds of thousands of measured quantities. Low-sample size (<40) can be handled as well; while the variance in predictive performance can be large, the user can still gauge the clinical utility of the results by the reported confidence intervals. In general, no statistical knowledge or expertise is required, nor any computer programming abilities. However, some basic statistical knowledge is still necessary to fully interpret all reported metrics and graphs. Special emphasis is placed on facilitating biological interpretation and clinical translation^10^. To this aim, results are complemented by (i) confidence intervals of performance to convey clinical utility, (ii) ROC curves that allow one to choose the optimal trade-off between false positive and false negatives for clinical operational use, (iii) visualization of all identified signatures and biomarkers to understand the underlying biology and design feasible laboratory assays, (iv) metrics of marker impact to the predictive power (importance weighting) to optimize the cost-benefit trade-off of including all or some biomarkers in the model, (v) a visualization of the role of each marker to the prediction (Individual Conditional Expectation or ICE) plot to facilitate biological interpretation, (vi) scatter plots of predictions to identify possibly mislabeled data, (vii) interpretable models that trade-off predictive performance for human interpretability, and (viii) automatically generated text reports explaining the analysis that took place and summarizing the results. The claim is supported by hundreds of omics analyses performed as part of this paper, as well as by a use case reproducing an analysis published in Nature Medicine^23^. In this use case, we obtained models of quality comparable to the ones produced by human expert analysts, employing fewer markers with minimal human effort, no expert knowledge, and a few minutes of computational time. In addition, a few dozen papers with JADBio applications have already been published in the literature^36–65^.

While other AutoML platforms automate the production of predictive models, they often lack other important functionality for translational research (Table 1). We argue that the current view of AutoML is to automate the delivery of a model, rather than to provide the full range of information required for successful interpretation and application of the model. In our opinion, this perspective is better described by the terms CASH (combined algorithm selection and hyperparameter optimization) or HPO (hyper-parameter optimization)^66^ and not AutoML. AutoML should instead be a term that implies automation at a different level, ideally delivering all information and insight that a human expert would deliver. JADBio is a step towards this direction, although of course, still far from fully realizing this vision.

A major AutoML functionality of JADBio that is missing from other AutoML tools is feature selection (biosignature identification, biomarker discovery), i.e., identifying a minimal-size subsets of biomarkers that are jointly optimally predictive. Feature selection is often the primary goal of the analysis. The selected features lead to biological insights, cost-effect multiplex assays of clinical value, and identification of plausible drug targets. Feature selection is a notoriously difficult and combinatorial problem: markers that are not predictive in isolation (high unconditional *p*-value) may become predictive when considered in combination with other markers. The reverse also holds: markers that are predictive in isolation (low unconditional *p*-value), may become redundant given the selected features (high conditional *p-*value). JADBio embeds feature selection in the analysis process that scales to hundreds of thousands of features often encountered in (multi)-omics studies. GAMA and auto-sklearn do not attempt feature selection at all, while TPOT and TPOT-MDR do try feature selection methods, but they lead to the winning model only occasionally (11% and 2% of the times).

Even more challenging is the problem of multiple feature selection, i.e., identifying all multiple feature subsets of minimal size that lead to optimally predictive models. In the case-study above, there were three feature subsets identified in the FR cohort (Fig. 3a), of five microbe species each, that lead to equally predictive models. Each one suffices for predictive purposes. But, when feature selection is used for knowledge discovery, it is misleading not to report all three signatures. In addition, identifying all of them provides design choices to the engineer of a diagnostic assay. The multiple feature selection problem has so far received relatively little attention by the community with only a handful of algorithms available^67–69^. JADBio is the only tool offering this type of functionality. Prior research has shown that there are indeed multiple signatures present in molecular data^32^. Our results also corroborate this finding with the SES algorithm^69^ discovering more than 10 signatures in 59% of the analyses where it is employed in the optimal configuration.

Second, we claim that JADBio can significantly reduce the number of selected features (biomarkers) without compromising model quality in typical omics studies. This claim is supported by Fig. 4 and Supplementary Table 4, showing that when feature selection in JADBio is enforced (shown as JADBio-FS) the median AUC is decreased by only 0.0034 points with respect to the leading performing tool, namely TPOT-MDR, while the number of features selected is reduced by about ~4000 times. Interestingly, we note that Random Forests using the default settings can achieve predictive performance comparable with the results of much more sophisticated and computational-resource hungry CASH platforms on omics data. When predictive performance is the only goal in the analysis of an omics dataset, running solely Random Forests may suffice.

The third claim is that JADBio provides accurate estimates of the out-of-sample performance with no need of an independent holdout set. This is supported by the results in Fig. 5 and Supplementary Table 6. The statement has the following serious ramifications: no samples need to be lost to estimating performance by the user. The final model is trained on all samples. JADBio automatically handles the estimation of performance and its uncertainty (confidence intervals). To clarify, we claim that the user does not need to hold out a separate test set to statistically validate the final model. This is of particular importance in omics datasets, often including a small number of patients due to rare conditions and cost. We would like to add the disclaimer however, that the estimates are valid only within the same operating environment. If batch effects or other distributional changes are possible, a separate external validation set should be employed. This property of JADBio is necessary for full automation of analysis. Instead, the estimates by GAMA, TPOT, and auto-sklearn are shown to systematically overestimate (Fig. 5), arguably because such tools do not employ experimentation protocols devised for performance estimation on the training set.

What are the key ideas that enable JADBio to overcome key challenges? The use of an AI decision-support system that encodes statistical knowledge about how to perform an analysis makes the system adaptable to a range of data sizes, data types, and user preferences. An automated search procedure in the space of appropriate combinations of algorithms and their hyper-parameter values, trying thousands of different machine learning pipelines (configurations) automatically optimizes choices. Protocols that estimate the out-of-sample predictive performance of each configuration, particularly the Generalized Cross Validation (see Methods) suitable for small sample sizes *reduces the uncertainty* (variance) of estimation. Treating all steps of the analysis (i.e., preprocessing→ imputation→ feature selection → predictive modeling) as an atom, and cross-validating configurations rather than just the final modeling step avoids overestimation. A statistical method for removing the performance optimism (bias) due to trying numerous configurations (BBC-CV) is also necessary to avoid overestimation. The use of a feature selection algorithm that scales up to hundreds of thousands of biomarkers, suitable for small sample sizes allows the identification of multiple statistically equivalent biosignatures (SES algorithm)^69^.

Lastly, notice that the final model suggested for clinical use is constructed on all samples so that no samples are lost to estimation. On average, the model fit on all data will be the most predictive. So, how is its performance estimated, since we have no data left for estimation? JADBio cross-validates the whole process that produces final model and estimates the performance of this process. This signifies a shift in estimation perspective: it is the model-producing method that is evaluated, not a specific model instance.

There are numerous limitations in the study. First, the conservative estimation of out-of-sample performance has only been shown for a random splitting of the data into training and test set. While this is standard in machine learning practice, it does enforce that the joint distribution of both the predictors *X* and the outcome *Y* remains the same between the train and the test set. This may not be true, however, in clinical applications where there is inter-tumor heterogeneity. For example, different tumor subtypes may be more prevalent in the model’s operational environment than in the training data. Thus, clinical expertise must be applied before trusting the model estimates based on the assumption above^70^.

The scope of automation and experimental validation concerns low-sample, high-dimensional transcriptomics, methylomics, and metabolomics datasets and binary outcomes. Transfer of the conclusions to other types of molecular data (e.g., proteomics, single cell, genetic) and outcome types needs further study. Note however, that JADBio has already been successfully applied to datasets with much larger samples^36,50^. The scope of the experiments is limited with regards to imbalanced data, as the most extreme class distributions tried contain at least 25% samples in the minority class.

The automation architecture presented does not include preprocessing steps of the raw molecular data and signal. Features included in the biosignatures are selected on the basis of statistical criteria, and the interpretation of their biological relevance must be separately assessed, if needed. JADBio does not encompass the full range of modern machine learning tasks, such as clustering, representation learning, or causal discovery, to name a few. Predictor types do not include free medical text or measurements over time (longitudinal data). Prior medical knowledge in the form of biological and medical ontologies or pathways is not considered during the analysis (however, see our recent work^71^ that converts gene expressions to pathway scores). Datasets are analyzed independently of all other datasets publicly available; recent research directions try to consider datasets in their totality^72^ instead. Biosignatures identification and predictive modeling are just one of the steps of a long process in bringing new diagnostic or prognostic tests to the clinic. Propaedeutic actions include, but are not limited to, devising a meaningful study design and sampling procedures, while follow up steps include independent validation studies, the development of tests suitable for the clinical practice, and cost-benefit analyses.

In conclusion, we advocate that JADBio’s AutoML approach could accelerate precision medicine and translational research. Specifically, it could facilitate the discovery of novel biosignatures and biomarkers leading to new biological insights, precision medicine predictive models, drug targets, and non-invasive diagnostics in cancer^42^ or other conditions^53,60^.

## Methods

### Benchmark datasets used in the quantitative comparative evaluation

We collected data from repositories offering datasets with case-control binary outcomes and include both molecular profiles and curated meta-data (i.e., study design information). BioDataome^73^ is such an online repository with *transcriptome* (both *microarray* and *RNA-seq*) and *epigenetics* (methylation array) datasets. BioDataome uniformly processes and automatically annotates datasets from the Gene Expression Omnibus database (GEO)^1^ and the RECOUNT database^2^. It uses a text-mining pipeline for automatically separating profiles in controls and cases, if applicable, obtaining a dichotomous target for prediction. This automatically assigned status (cases vs. controls) was chosen as the binary outcome of interest. Metabolomics datasets were obtained from the Metabolomics Workbench^3^, a repository funded by the “NIH Common Fund Metabolomics” initiative. We manually identified a suitable dichotomous prediction target for each metabolomics dataset out of their respective meta-data. As inclusion/exclusion criteria we selected all studies from BioDataome and Metabolomics Workbench with at least 40 samples for which a binary outcome could be identified. We also require at least ten samples for each class. For BioDataome we focused on the GPL570 (Affymetrix Human Genome U133 Plus 2.0 Array) and GPL13534 (human methylation 450k BeadChip array). These criteria lead to the selection of 271 transcriptomics microarray datasets (54675 measurements each, 31630 samples in total), 23 methylation datasets (485512 measurements each, 2322 samples in total), 23 RNA-seq datasets (59037 measurements each, and 2165 samples in total), and 43 metabolomics datasets (1491 measurement on average, sd. 4909, and 3792 samples in total).

The datasets are related to 125 diseases or phenotypes, with cellular proliferation diseases (i.e., different types of cancers) being the most represented group with 154 datasets. Other illnesses include chronic obstructive pulmonary disease, psoriasis, and mental health diseases. In total, we analyzed 39909 samples (molecular profiles), corresponding to ~2.98 × 10^9^ data values, with each profile measuring between 15 and 485512 molecular quantities (variables, features), 76063 on average. Supplementary Data 1 lists all datasets and respective characteristics.

Normalization of microarray gene expression data was performed in BioDataome using the single-channel array normalization (SCAN) algorithm^74^. SCAN normalizes each array *independently*, ensuring that measurements from profiles in the test sets do not affect the preprocessing of profiles in the training sets; thus, there is no information leakage from the test sets during cross-validation to the estimation of performance from the training set. Count values for RNA-seq data were downloaded as prepared by the RECOUNT repository. Each sample was then *independently* normalized for library size (estimated as the sum of all its reads) and log2-transformed. More sophisticated methods do exist both for library size normalization and variance-stabilizing transformation (see for example the approaches provided in the DESeq2 R package^75^), but they do not preprocess samples independently and would require special treatment, i.e., to be incorporated within the cross-validation procedure. Background correction and normalization of methylation data was carried out with the minfi R package^76^, with beta values used for all subsequent analyses^77^.

### AutoML and CASH platforms included in the evaluation

We run JADBio by optimizing four different criteria, namely Performance (JADBio-P), Interpretability (JADBio-I), Feature Selection (JADBio-FS), and Aggressive Feature Selection (JADBio-AFS). These different settings lead to the configuration and corresponding model that emphasize performance, interpretability (selecting only among humanly interpretable models), and number of features selected, respectively. JADBio-AFS runs feature selection algorithms that perform more aggressive feature selection and at the expense of predictive performance on average. Only one run for JADBio is required; optimization for different preferences is performed post-analysis without having to rerun the analysis.

We compare JADBio against auto-sklearn^17^, TPOT^18^, TPOT-MDR^19^, GAMA^20^, AutoPrognosis^21^, and Random Forests^22^. Auto-sklearn is among the state-of-art libraries for automated machine learning, winner of both international AutoML challenges competed so far^78^. We use the Random Forest algorithm^22^ with default parameters as implemented in the scikit-learn library^31^. The latter is not considered an AutoML platform, but it is used as a baseline method to gauge the benefits of AutoML tools. TPOT-MDR is a recently introduced variant of TPOT that searches over a series of feature selection and Multifactor Dimensionality Reduction (MDR) models specifically to address high-dimensional, genomic data^19^.

### Evaluation experimental design

Each dataset was split in half in terms of samples in a stratified way (i.e., the distribution of classes was kept about the same in the two halves as in the original). All tools and variants were executed on the first half to produce a model and a training performance estimate and applied on the second half to obtain a test performance estimate (holdout performance). The roles of each half are then reversed leading to a total of 360 × 2 = 720 runs for each platform. GAMA, TPOT, TPOT-MDR and auto-sklearn all require an indicative time limit for the completion of the analysis. This time limit was set to the maximum between 1 h and the termination time of JADBio on the same task. Despite the time-limit these tools may still take longer; runs that exceeded twice the time limit were forcefully terminated. Each run was performed in parallel on 20 CPUs, using 256 GB of RAM (400 GB for methylation datasets). AutoPrognosis does not run in parallel, thus we run it on a single CPU giving 20 times the time used by JADBio as time limit. Random Forest was not given any specific time limit and was also run on a single CPU. Finally, and most importantly, all hyper-parameters of all tools were set to their default values. The only exception was the performance metric: AUC was employed by all tools for identifying the best-performing model, regardless of their default metric, to ensure a fair comparison.

For this computational experiment, 79,603,051 predictive models were constructed by JADBio alone. It was not possible to extract the number of models trained by the other tools from the logs of the other platforms.

### Permutation-based statistical tests for comparing predictive performances

We assess the statistical significance of the median difference in holdout AUC between two AutoML tools *T*_1_ and *T*_2_ through a permutation-based test. Each tool successfully completed a subset of the 720 runs, namely $S_{T_1}$and $S_{T_2}$, with *S*_*c*_ indicating their intersection, i.e., the runs both tools completed. For each run $i \in S_c$, both tools *T*_1_ and *T*_2_ produced a holdout AUC, namely *AUC*_*i*1_ and *AUC*_*i*2_, which are computed on the exact same holdout set and are thus directly comparable. We define the median holdout AUC difference $M_{T_1 - T_2}$ between tools *T*_1_ and *T*_2_ as the median of the vector $\left[ {AUC_{i1} - AUC_{i2},\;\forall \;i \in S_c} \right]$. Under the null hypothesis that the two tools have equal predictive capabilities, for each run *i* the performance of the two tools should be exchangeable. We thus estimate the null distribution of $M_{T_1 - T_2}$ by randomly exchanging each *AUC*_*i*1_ and *AUC*_*i*2_ with probability 0.5 and recomputing the median of the differences. By repeating this process 10,000 times, we obtain the distribution $[ {M_{T_1 - T_2}^1, \ldots ,M_{T_1 - T_2}^{10000}}]$. A two-tailed p-value is computed by quantify the proportion of $abs( {[ {M_{T_1 - T_2}^1, \ldots ,M_{T_1 - T_2}^{10000}}]})$ larger or equal than $abs\left( {M_{T_1 - T_2}} \right)$, where *abs* indicates taking the absolute value.

A similar test is used for assessing whether the bias in performance estimation for each tool is different from zero in a statistically significant way. For each tool *j* and run *i*, our experimentation protocol computes both the performance on the holdout set, namely *AUC*_*ij*_, and an estimate derived from the training set, namely *TRAUC*_*ij*_. The corresponding bias is defined as $b_{ij} = AUC_{ij} - TRAUC_{ij}$, and the median bias for tool *j*, namely *B*_*j*_, is the median value of the vector $\left[ {b_{ij},\;\forall \;i \in S_{T_j}} \right]$. If the null hypothesis that the bias is symmetrically centered around zero is true, it is then possible to randomly change the sign of each *b*_*ij*_ with probability 0.5. We applied this procedure 10,000 times, generating the null distribution $[ {b_{ij}^1, \ldots ,b_{ij}^{10000}}]$. A two-tailed p-value is then computed by quantifying the proportion of $abs( {[ {b_{ij}^1, \ldots ,b_{ij}^{10000}}]})$ that is larger or equal than *abs*(*b*_*ij*_).

### The JADBio architecture

**Fig. 6:**
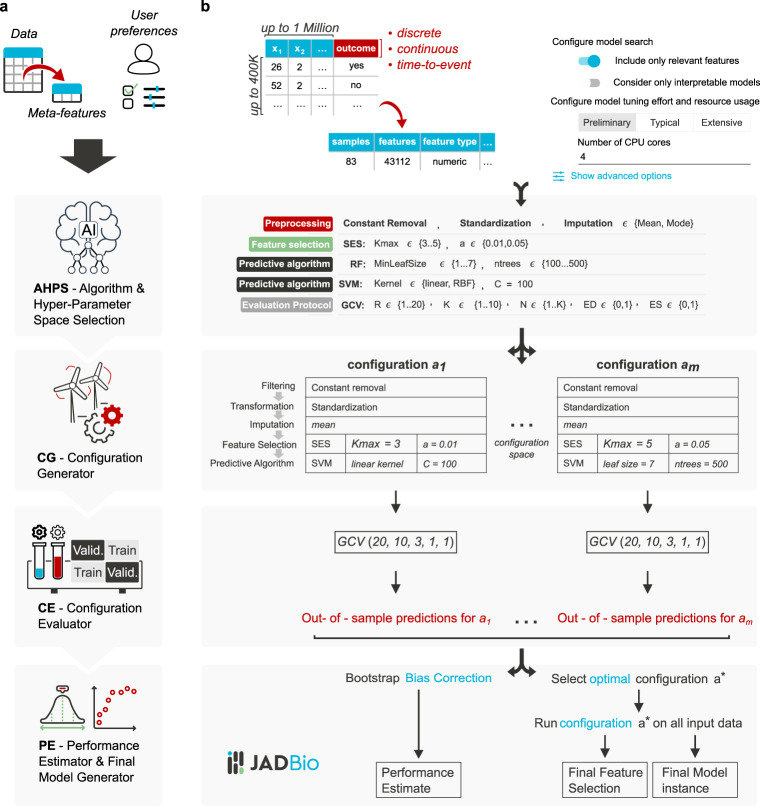
JADBio architecture. Panel **a** visualizes the architecture at a high-level, while panel **b** visualizes the details. The Algorithm and Hyper-Parameter Space selection system (AHPS), analyzes the dataset meta-features (characteristics of the data such as sample size) and user preferences. Based on them, it selects the appropriate combinations of algorithms and hyper-parameter values to try which form the configuration space to explore. It also decides the hyper-parameters of the generalized cross validation (GCV) algorithm, i.e., the protocol for estimating each configuration’s performance. Based on these decisions, the Configuration Generator (CG) instantiates the configurations to be evaluated. The Configuration Evaluator (CE) identifies the winning configurations using generalized cross-validation (GVC), i.e., by repeatedly applying each configuration on different partitions of the data (“Train” boxes) to train a model and estimating performance on the remaining data (out-of-sample predictions, “Validation” boxes). The Performance Estimator and Final Model Generator (PE) produces the final model and its performance. It pools together all out-of-sample predictions during cross-validation and feeds them to the Bootstrap Bias Correction algorithm. This is to remove the bias due to trying multiple configurations. The final model is produced on all available data using the winning configuration.

Figure 6 illustrates JADBio’s architecture. JADBio accepts data in a two-dimensional table format; rows corresponding to samples and columns to measured features (a.k.a. biomarkers, variables, quantities, predictors). One of the table columns must define the outcome of interest. JADBio handles dichotomous (binary classification), nominal (multi-class classification), continuous (regression), and time-to-event (e.g., survival analysis, right-censored outcome) type of outcomes. JADBio then analyzes the dataset fully automatically. A JADBio analysis is a four-stage procedure consisting of the Algorithm and Hyper-Parameter Space selection (AHPS) system, the Configuration Generator (CG), the Configuration Evaluator (CE), and the Performance Estimator and Final Model Generator (PE). Their role and connections are explained below.

### Algorithm and hyper-parameter space selection (AHPS)

Initially, the input data and user preferences are processed by the Algorithm and Hyper-Parameter Space selection system (AHPS). AHPS is an AI Decision Support System that decides which algorithms for preprocessing, imputation of missing values, feature selection, and modeling to combine (when images are analyzed, a feature construction step is also included; details are out of scope of this paper). It also decides on the hyper-parameter values to try. Essentially, each combination is a unique ML pipeline that takes a dataset and produces a predictive model; we call it a configuration. The goal is of course, to identify the optimal configuration that leads to the best possible model instance. An example of a configuration is “impute missing values with their mean, run the Statistically Equivalent Signatures algorithm for feature selection with hyper-parameter values *a* = 0.05, and maxk = 3, then run a Support Vector Machine with linear kernel and cost hyper-parameter *C* = 100”. Typically, the number of possible configurations ranges between a few tens to a few thousand.

AHPS represents knowledge using two methodologies: (a) a rule-based AI Decision Support. A simple example of a rule is “when sample size is <100, try the SES algorithm with hyper-parameter alpha (level of significance) in the set {0.01, 0.05, 0.1}”. The result of all these rules determines the configuration space, i.e., all configurations that are reasonable to apply to the problem at hand. The rules have been determined by our personal expert experience and preliminary experimentation. (b) Additional knowledge and rules are induced from results of algorithms on past analyses. This technique is called meta-level learning. Specifically, meta-level learning is the process of analyzing the results of configurations on previous datasets, based on the meta-level features of the data (e.g., sample size, dimensionality, feature type, missing values percentage, etc.) to build meta-level models^79^. These models predict which configurations are expected to exhibit high performance on a new unseen dataset. As a result, non-promising configurations are filtered out before execution to achieve computational savings without reducing expected performance. Using meta-level learning, JADBio is expected to keep self-improving with usage.

Finally, the AHPS decides on the configuration evaluation protocol, i.e., how to estimate the performance of each configuration and select the winner to produce the final predictive model. All protocols are out-of-sample protocols, i.e., they train a configuration with only a portion of the data and evaluate the resulting model on the remaining held-out data (a.k.a. validation data). The predictions of a model instance produced by a configuration on samples not included in the training of the model are called out-of-sample predictions. These protocols are explained in more detail at JADBio algorithms section.

### Configuration generator (CG) and configuration evaluator (CE)

Next, the Configuration Generator (CG) instantiates the set of configurations to try within the space of choices output by AHPS. Each configuration is fed to the CE for evaluation and selection of the winner. We should note that CE evaluates each configuration as an atom: the preprocessing (imputation, normalization, standardization), feature selection, and modeling algorithms are jointly evaluated as one procedure. This avoids a common methodological mistake where preprocessing, and feature selection are first applied on the complete dataset and only the last step of modeling (e.g., the decision tree algorithm) is cross-validated^80^. The error could lead to significant overestimation of performance, overfitting, and invalidation of results: an eye-opening pedagogical experiment is presented at page 245 of Hastie, Tibshirani, and Friedman’s book^81^, where the true error rate is 50% but the estimated error is only 3% if one applies first the feature selection step on all data and only then cross-validates only the learning algorithm.

### Performance estimator and final model generator (PE)

A typical requirement in predictive modeling is to separate the data, a priori, in two parts; one to use for generating (training) the final model, and another for estimating (testing) its performance. Assuming, however, that a configuration produces better performing models as sample size increases, training the winning configuration on the full dataset is expected to produce the best possible model. For this reason, JADBio applies the winning configuration as determined from the CE on the full dataset to generate the final model instance and final selection of features. This means that JADBio will not lose any samples to estimation of performance.

This begs the question however, how is performance estimated for the final model? The answer is that JADBio does not directly estimates the performance of the final model; instead, it estimates the performance of the configuration (i.e., of the methodology) that produces the final model. Using generalized cross validation, the CE has already estimated the out-of-sample performance of the models produced by the winning configuration. Unfortunately, this estimate is optimistic and should not be returned to the user. This is because when selecting the best-performing configuration among many, it is likely that it achieved this performance because it got “luckier” than average on the specific validation sets. This phenomenon is better known as the “multiple comparison in induction algorithms problem”^12^, which is related to the “winner’s curse” in statistics^11^. The overestimation has been proved theoretically, but also shown empirically in Tsamardinos et al. (2018)^28^. To correct for optimism, JADBio employs a recently developed^28^ protocol, namely Bootstrap Bias Corrected CV (BBC-CV) to adjust performance for multiple tries. BBC requires the out-of-sample predictions on each sample for each configuration tried and stored by CG. To apply BBC, one needs to store the out-of-sample predictions made by all configurations on all validation subsets during cross-validation. These are indicated as “out-of-sample predictions for *a*_*i*_” in Fig. 6.

### JADBio algorithms

A configuration in JADBio comprises of several types of algorithms, namely algorithms for data preprocessing, data transformation, data imputation, feature selection, and predictive modeling. In addition, JADBio employs algorithms implementing the AHPS, the CE and the PE. Table 2 lists all algorithms employed by JADBio. Each algorithm type is now explained in detail.

**Table 2:** Algorithms used by JADBio depending on the outcome of interest.

Algorithm	Used for	*Class*.	*Regr*.	*Surv*.	*Implemented by*
AI Decision Support	Knowledge Representation	+	+	+	in-house
MLL Configuration Filtering	Meta-level learning	+	+	+	in-house
Mean/Mode Imputation	Preprocessing	+	+	+	in-house
Standardization	Preprocessing	+	+	+	in-house
Lasso^82^	(single) Feature Selection	+	+	+	glmnet^91^
SES^69^	(multiple) Feature Selection	+	+	+	in-house
Decision Trees^84^	Predictive modeling	+	+	+	in-house
Ridge Regression^85^	Predictive modeling	+	+		in-house
Random Forests^22^	Predictive modeling	+	+		in-house
Support Vector Machines^86^	Predictive modeling	+	+		libsvm^86^
Cox Regression^87^	Predictive modeling			+	in-house
Random Survival Forests^88^	Predictive modeling			+	in-house
Generalized Cross-Validation^28^	Performance estimation	+	+	+	in-house
Early Dropping^28^	Configuration space search heuristic	+	+	+	in-house
Early Stopping	Configuration space search heuristic	+	+	+	in-house
BBC-CV^28^	Performance correction	+	+	+	in-house
BBC4ROCs	Threshold optimization	+			in-house

### AI decision support and meta-level learning algorithms

These algorithms implement the AHPS component. They determine the configuration space and the configuration evaluation protocol. The Decision Support system is implemented by defining rules on top of an ontology for machine learning concepts that is defined in OWL. The Meta-Level Learning (MLL) algorithm analyzes past runs of configurations to induce new rules for configurations that should be filtered out and not tried in an analysis, given the meta-level features of a dataset. A full description of this system is out of scope of this paper, as its description and validation experiments are extensive.

### Data preprocessing algorithms

JADBio performs mean and mode imputation of the missing values for the continuous and categorical features, respectively. Zero variance (or close to machine epsilon) features are removed. Continuous features are standardized to zero mean and standard deviation of 1. Categorical features are treated using a 1-hot-encoding.

### Feature selection algorithms

Feature selection algorithms try to discover the minimal size, optimally predictive feature subset. JADBio employs two such algorithms at the moment, namely, LASSO regularized regression^82^ and a (independence) test-budgeted version of the Statistical Equivalent Signatures (SES^69^). SES is arguably more appropriate for small samples sizes and tends to return smaller feature subsets at the expense of predictive performance. It is inspired by causal model and the theory of the Markov Blanket. Lasso is arguably the most common feature selection algorithm and solves a global optimization problem. It tends to perform better for larger sample sizes but selects features. A major difference between the two algorithms is that SES returns multiple feature subsets (signatures) that lead to statistically indistinguishable predictive performances. The standard Lasso does not (however, we have extended it to return multiple equivalent solutions^37^ but it is not yet incorporated into JADBio). The importance of returning multiple equivalent feature subsets and the difference of feature selection from standard differential expression analysis is elaborated in the Discussion section.

### Predictive modeling algorithms

JADBio incorporates a collection of model families that were chosen based on (a) ease of interpretation and (b) their performance on large comparative evaluations^83^. In the latter work the authors ask: “Do We Need Hundreds of Classifiers to Solve Real World Classification Problems?” to which the answer is negative: a few, carefully selected algorithms, tuned correctly often suffice to achieve optimal performance. Specifically, depending on the type of outcome (Table 2), JADBio tries Decision Trees^84^, Ridge Logistic Regression^85^, Random Forests^22^, (linear, polynomial, and RBF) Support Vector Machines^86^, Cox regression^87^ and Random survival forests^88^ as well as the baseline algorithm that classifies to the most prevalent class. However, we do note that this list is only indicative, as we constantly keep enriching the algorithmic arsenal of JADBio. Most implementations of these algorithms are in-house and optimized for speed and quality. In addition, JADBio allow the user to customize an analysis with additional publicly available algorithms through its API.

### Configuration evaluation algorithm for performance estimation

The configuration evaluation algorithm is an essential component of JADBio and a major contribution of this paper. It affects predictive performance as well as computational time. JADBio does not apply a one-size-fits all protocol, e.g., a 10-fold cross validation (CV) on all datasets. If it did, it would not scale up to hundreds of thousands of samples, scale down properly to tiny samples, or correctly handle class imbalance. Instead, JADBio chooses the optimal protocol among the possible instantiations of a single configuration evaluation algorithm that we call Generalized Cross Validation or GCV (not to be confused with the homonym algorithms for spline smoothing^89^). GCV can emulate several protocols by appropriately choosing its hyper-parameters, which are special cases of GCV, such as the holdout (train-validate) protocol, K-fold cross-validation (CV), the incomplete CV^28^, the repeated CV^28^, and others. GCV is a stratified, R-repeated, K-fold, N incomplete CV that accepts the values of *R*, *K*, and *N* as hyper-parameters. We now explain these in turn.

CV is a standard technique where the sample size is partitioned to *K* folds of approximately equal size; a configuration produces a model instance based on all sample folds but one, and an out-of-sample performance estimate is produced on the held-out fold. The predictions of the model on the held-out fold are saved as out-of-sample predictions of that configuration. The performance estimate of the configuration is the average performance over all folds. There is an important subtle point in this methodology: it is the performance of a configuration that is being estimated, not the performance of a specific model instance. Thus, even though the final model returned by JADBio is trained on all samples, the final estimate stems from estimating the performance of the configuration that produced the model instance, and not the performance of the model instance itself.

Stratification (based on the class in classification problems) implies that each fold in cross-validation follows approximately the same distribution of classes as the un-partitioned dataset^33^. R-repeated CV implies that the cross-validation procedure runs *R* times with different partitions to folds to reduce the variance in the estimation due to the specific partitioning and to tighten the confidence intervals^28^. The reduced uncertainty in performance estimation also implies that the true best-performing configuration is selected with higher probability, thus repeating the CV also improves predictive performance. By *N* incomplete CV we denote a CV in which only N iterations out of the *K* are performed in the last repeat of the CV. Let us present some examples. For large sample sizes, a 10% hold out protocol can be emulated by setting *R* = 1, *K* = 10, *N* = 1: the dataset will be partitioned once (*R* = 1) to 10 folds (*K* = 10), out of which only 1 (*Ν* = 1) will serve as a test fold. Setting *R* = 1, *K* = 10, *N* = 3 would train three models for each configuration on 90% of the data each improving the performance estimates. To perform a standard 10-fold CV, we would set *R* = 1, *K* = 10, *N* = 10. For tiny sample sizes, we would use *R* = 10, *K* = max, *N* = max, i.e., repeat ten times the CV procedure with as many folds as possible. JADBio sets the maximum number of folds to the number of samples in the rarest class, so that each fold contains at least one sample from each class. This requirement avoids some of the problems of standard leave-one-out CV^90^. The values of *R*, *K*, and *N* are decided by the AHPS system to reach a certain size of out-of-sample predictions, considering the total sample size, the samples per class, the imbalance between classes, and the number of censored outcomes in a time-to-event analysis. GCV is also equipped with two heuristic algorithms, described below.

### Configuration space search heuristics

After the AHPS decides which configurations will be tried, the model space is explored. Currently, JADBio employs generic grid search for hyper-parameter optimization. Despite this being a simple static strategy, JADBio equips GCV with two heuristics to reduce computations without compromising quality. The Early Dropping heuristic^28^ stops cross-validating a given configuration once it determines that it will not be the winning one with high probability. The latter is determined by a statistical test. The Early Stopping heuristic stops CE from repeating CV with different partitions early. It tracks the shrinkage of the confidence intervals during each repeat of the CV and when there is no progress achieved by the current repetition it terminates GCV. In the above example where we set *R* = 10, *K* = max, *N* = max, this heuristic may stop CE at the 5th repeat and R may never reach ten. Early Dropping requires a minimum certain sample size for the statistical tests, so it is only applied when AHPS determines it is safe to do so.

### Performance correction algorithms

For estimating the performance of the final model, JADBio employs the Bootstrap Bias Correction estimation algorithm or BBC^28^. BBC is conceptually equivalent to adjusting *p*-values in hypothesis testing for the fact that many hypotheses have been tested; similarly, BBC adjusts prediction performances for the fact that many configurations (combinations of algorithms) have been tried. The main idea of BBC-CV is to bootstrap the configuration selection strategy on the out-of-sample prediction matrix produced during the cross-validation. In this way, BBC-CV removes the bias due to the multiple tries, hence, removing the need for an external test set, provided it is applied on populations with the same distribution as the training data. BBC-CV is one order-of-magnitude more efficient than the previous protocol that corrects for multiple tries, namely the nested cross-validation^80^. The BBC-CV protocol can output, with no additional computational overhead, the probability distribution of the expected performances and their 95% confidence intervals.

We would like to note that JADBio performs a BBC correction on all performance metrics it reports unless it is explicitly specified. Particularly for binary classification problems, we have developed the *BBC4ROCS* algorithm that estimates the sensitivity, specificity, F1, accuracy, and other threshold-dependent metrics for ten different classification thresholds. These thresholds correspond to the respective quantiles of the out-of-sample predictions of the best model so that their distribution is divided into areas of equal probability. The metrics are then BBC corrected leading to adjusted-for-multiple-tries ROC curves along with their confidence intervals on the ROC curve. The CIs span both dimensions of the ROC space, namely False Positive Rate and True Positive Rate, creating a CI “cross” pattern. This allows the user to select the classification probability threshold that leads to the desired trade-off between sensitivity and specificity of the model, having corrected for multiple tries.

### Methods for post-analysis explanation, visualization, and model interpretation

Individual Variable Importance Plots: the purpose of this plot is to assess the added value of each selected feature. Individual variable importance measures the effect in predictive information when a single feature (variable) is removed. For each variable in turn, individual variable importance is computed as the ratio between the resulting cross-validated performance when the variable is removed and the cross-validated performance obtained on the original dataset; in both cases, the winning configuration is employed to build the models. To be more precise, a variable is not removed from the data, but its values are permuted instead, thus ensuring that it carries no predictive information. Virtually removing the variable through permutation, instead of removing it completely during cross-validation ensures that the dimensionality of the problem remains the same in both cases and that the best identified hyper-parameter values for all algorithms do not need to be adjusted for differences in the dimensionality of the learning task. The permutation technique is conceptually similar to the calculation of importance weighting in Random Forests^22^.

Individual Conditional Expectation (ICE) plots: these plots visualize the effect of a given variable on the predictions provided by a specific model. More in detail, let assume that a dataset *D* is composed by several samples (e.g., patients), $D_i,\;i = 1 \ldots n$. Each sample in turn is measured over a set of variables $X_j,\;j = 1 \ldots p$. This means that $D_i = \{ X_1 = x_{i1},\;X_2 = x_{i2}, \ldots ,X_p = x_{ip}\}$, or $D_i = \{ x_{i1},\;x_{i2}, \ldots ,x_{ip}\}$ for short, where $x_{i1},\;x_{i2}, \ldots ,x_{ip}$ are the actual numerical or categorical values making up the *D*_*i*_ sample. A model *f* is able to provide predictions (e.g., the probability *pr*_*k*_ of belonging to a class *k*) for any sample: $pr_k = f\left( {D_i} \right) = f\left( {\left\{ {x_{i1},x_{i2}, \ldots ,x_{ip}} \right\}} \right)$.

The ICE plot for a variable *X*_*j*_ is built by first producing a line for each sample *D*_*i*_. On the *x*-axis this line spans all the values that *X*_*j*_ assumes in the dataset, while the *y*-axis values of this line are produced by repeatedly applying *f* on *D*_*i*_, taking care each time to vary the value of *X*_*j*_. In formulae, $f\left( {\left\{ {x_{i1},x_{i2}, \ldots ,X_j = \chi , \ldots ,x_{ip}} \right\}} \right)$, where *χ* varies across all the values *X*_*j*_ assumes in the dataset. Once a line is produced for each sample, the ICE plot is easily obtained by averaging all lines in order to obtain the mean behavior of the predictions as *X*_*j*_ varies. The 95% confidence intervals are computed by taking the corresponding percentiles across all lines. The method was first described by Goldstein and co-authors^30^. The ICE plots convey useful information. The user can gauge whether observing an increased value of a variable *X*_*j*_ results in higher or lower probability for a specific outcome. It is possible for the ICE curves to be not monotonic, e.g., as a drug dosage increases, the probability of successful treatment increases and then decreases again, if the drug dosage becomes toxic. Also, the variance (confidence intervals) of the plot is quite informative: values of *X*_*j*_ with low variance imply that the corresponding predictions are highly affected by *X*_*j*_ and less by other variables. Conversely, values of *X*_*j*_ with high variance indicates that the model relies heavily on other variables as well in order to provide the corresponding predictions.

### Reporting summary

Further information on research design is available in the Nature Research Reporting Summary linked to this article.

## Supplementary information

Supplementary Material

Supplementary Data 1

REPORTING SUMMARY

## Data Availability

The paper is accompanied by the webpage https://jadbio.com/jadbio-extensive-evaluation-resource-page/. This page contains all the data used in the analysis. Alternatively, the datasets can be downloaded directly from Gene Expression Omnibus (microarray, RNA-seq and methylation data, https://www.ncbi.nlm.nih.gov/geo/) or Metabolomics Workbench (metabolomics data, https://www.metabolomicsworkbench.org/). All datasets are also available in BioDataome (http://dataome.mensxmachina.org/).

## Code availability

The companion webpage https://jadbio.com/jadbio-extensive-evaluation-resource-page/ contains the scripts to reproduce all figures, as well as the script used for running the AutoML tools that were contrasted against JADBio. JADBio is available as SaaS platform at http://JADBio.com. A free version is offered. JADBio also has a REST API interface and a Python wrapper of the API available at https://github.com/JADBio/JADBioPythonClient. Open-source implementations of two main algorithms in JADBio, namely the SES multiple feature selection algorithm, and the BBC-CV for adjusting CV performance for multiple testing, are available at the MXM R Package.
