# Supplementary material for: Just Add Data: automated predictive modeling for knowledge discovery and feature selection
Source: NPJ Precis Oncol. 2022 Jun 16;6:38. doi: 10.1038/s41698-022-00274-8 (PMC9203777; doi:10.1038/s41698-022-00274-8)
Supplement: Supplementary file 1 — Supplementary Material [file 41698_2022_274_MOESM1_ESM.docx]

­Just Add Data: Automated Predictive Modeling for Knowledge Discovery and Feature Selection

**Authors:** Ioannis Tsamardinos^1,2,3^*, Paulos Charonyktakis^1^, Georgios Papoutsoglou^1,2^, Giorgos Borboudakis^1^, Kleanthi Lakiotaki^2^, Jean Claude Zenklusen^4^, Hartmut Juhl^5^, Ekaterini Chatzaki^6,7^, Vincenzo Lagani^1,8^

^1^ JADBio Gnosis DA S.A., Science and Technology Park of Crete, GR-70013, Heraklion, Greece

^2^Department of Computer Science, University of Crete, Heraklion, Greece

^3^Institute of Applied and Computational Mathematics, Foundation for Research and Technology, Hellas, N. Plastira 100, Vassilika Vouton, Heraklion, GR-70013

^4^National Cancer Institute, National Institutes of Health, Bethesda, MD, USA

^5^Chief Executive Officer, Indivumed Group, Hamburg, Germany

^6^Laboratory of Pharmacology, Medical School, Democritus University of Thrace, Alexandroupolis, Greece

^7^Institute of Agri-food and Life Sciences, Hellenic Mediterranean University Research Centre, Crete, Greece

^8^Institute of Chemical Biology, Ilia State University, Tbilisi, Georgia

*To whom correspondence should be addressed: [tsamard.it@gmail.com](mailto:tsamard.it@gmail.com)

Supplementary Material

# Supplementary Figures


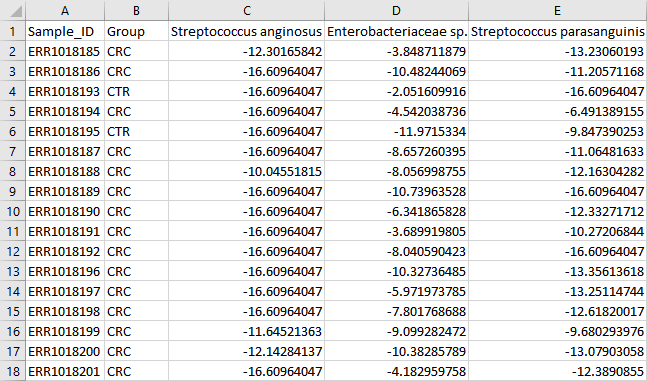

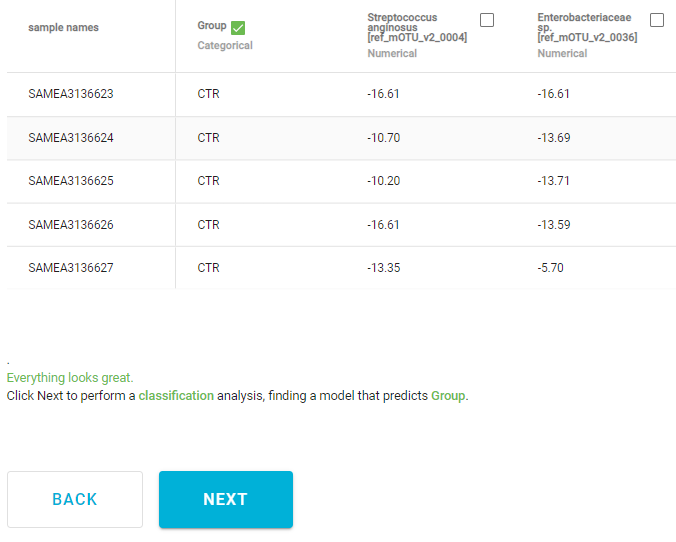

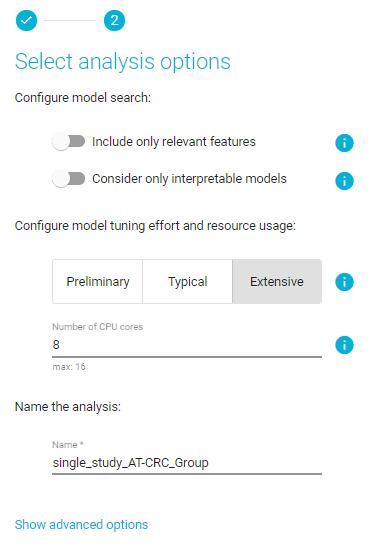


**a**

**b**

**c**

**d**


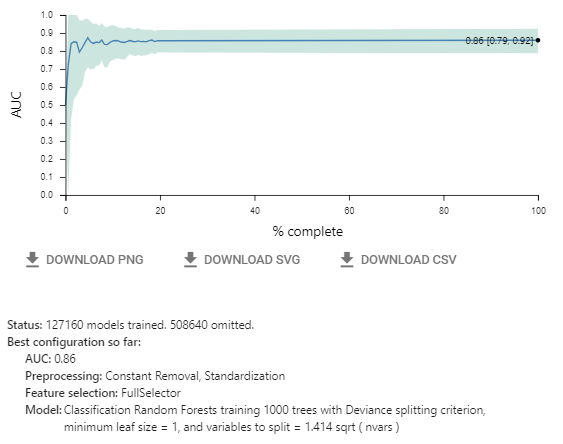


Supplementary Figure 1: Analyzing a dataset with **JADBio**. (a) A portion of the original data matrix in CSV format being drag-n-dropped to **JADBio**. The rows correspond to samples and the columns correspond to features. One of the columns should contain the outcome. In this case it is named ‘Group’ containing the disease status with categories colorectal cancer (CRC) and controls (CTR). (b) The user selects the column with the outcome to perform an analysis. (c) The user selects preferences, namely whether they wish to enforce feature selection (“Include only relevant features”), consider only interpretable models or not, how much computational effort to spend on tuning the choices to produce a final model, and how many CPU cores to allocate to the analysis. (d) Progress report of the analysis. An estimate of the best performance achieved so far and its confidence intervals, the percentage of analysis completed, and the steps (algorithms) of the currently winning configuration are shown.

Supplementary Figure 2: (a) precision recall curve for the winning model with Feature Selection trained on the CN cohort. The curve shows all trade-offs between recall (top x-axis) and precision (y-axis) for all different classification thresholds (bottom x-axis). By clicking on a circle, a corresponding threshold is selected (bottom x-axis). (b) Respective out-of-sample metric values and confidence intervals reported by JADBio as estimated after adjusting for trying numerous configurations (threshold = 0.488)

**a**


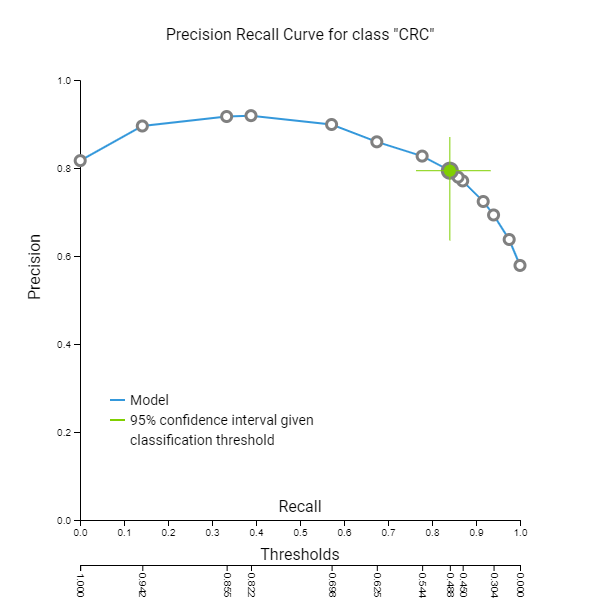


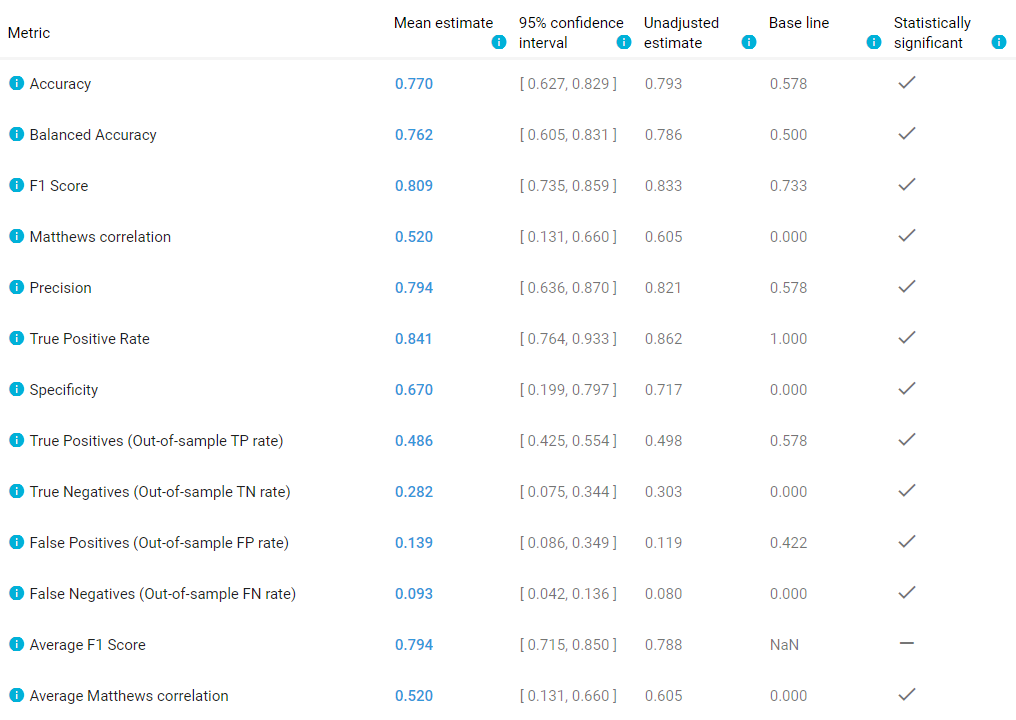


**b**


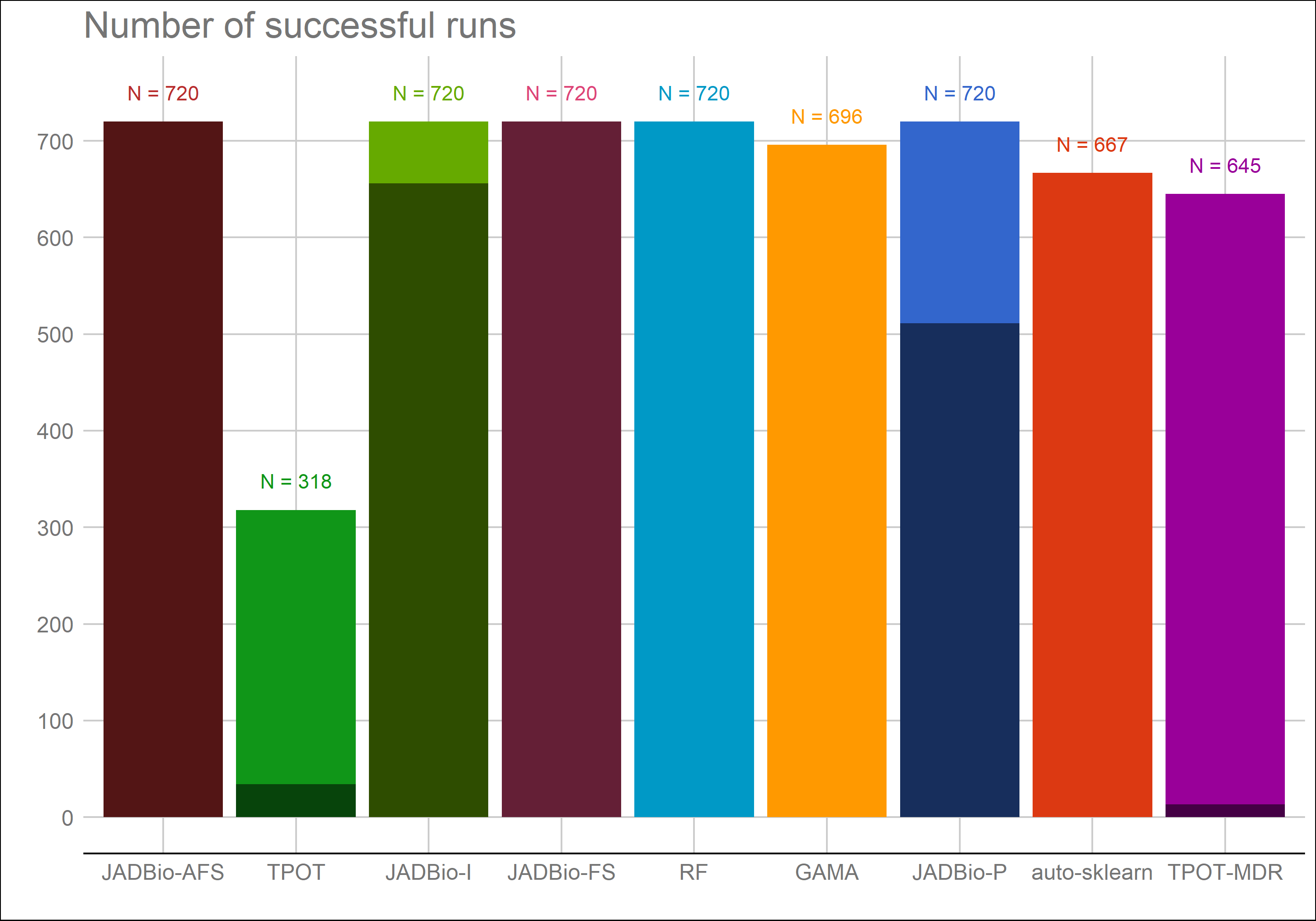


N = 13

N = 209

N = 511

N = 209

N = 64

N = 656

N = 284

N = 34

Supplementary Figure 3: Number of successfully completed runs for each tool. A darker shade within a column indicates the number of runs where feature selection was employed for building parsimonious models. The number of runs successfully completed is reported on top of each bar. Numbers within the bars indicate how many times feature selection is / is not selected by the tool. Feature selection was always adopted in all runs by JADBio-AFS and JADBio-FS. AutoPrognosis did not complete any run.


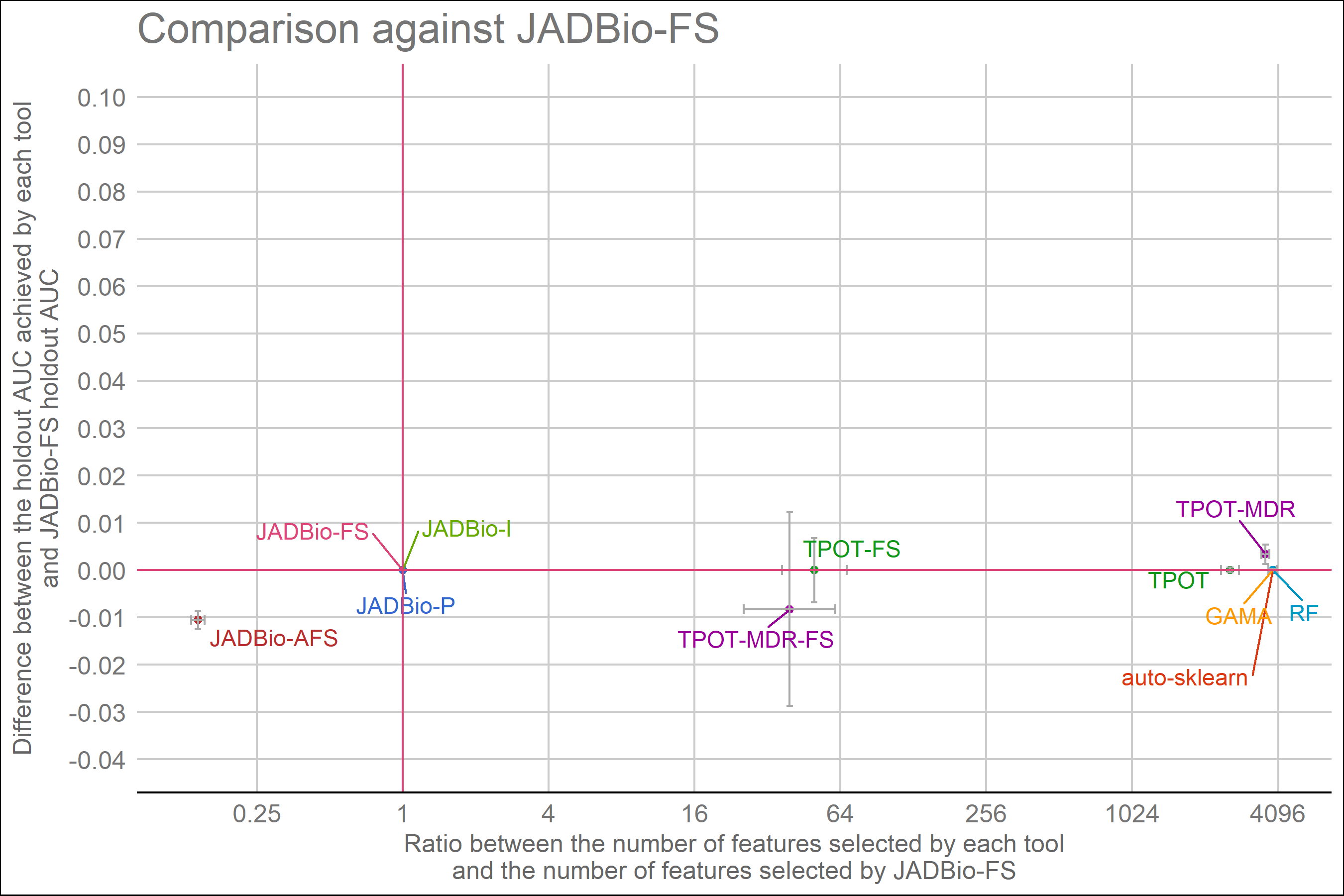


Supplementary Figure 4: Comparison between JADBio-FS and all other tools in terms of median dimensionality reduction and median performance gain. The dimensionality reduction with respect to JADBio-FS (x-axis) is computed as the ratio between the number of features selected by each tool and the number of features selected by JADBio-FS (log2 scale). Values larger than one indicate that the tool selects more features than JADBio. The performance gain is reported on the y-axis, and it is computed as the difference between the holdout AUC achieved by each tool and the holdout AUC achieved by JADBio-FS. Values larger than zero indicates that the tool performs better than JADBio-FS. Each point corresponds to a specific tool, and its coordinates are the median dimensionality reduction and the median performance gain across all runs that the specific tool has in common with JADBio-FS. Error bars are computed as median +/- median standard error. Cranberry colored lines denote baseline value, i.e., relative dimensionality reduction equal to one and performance gain equal to zero.


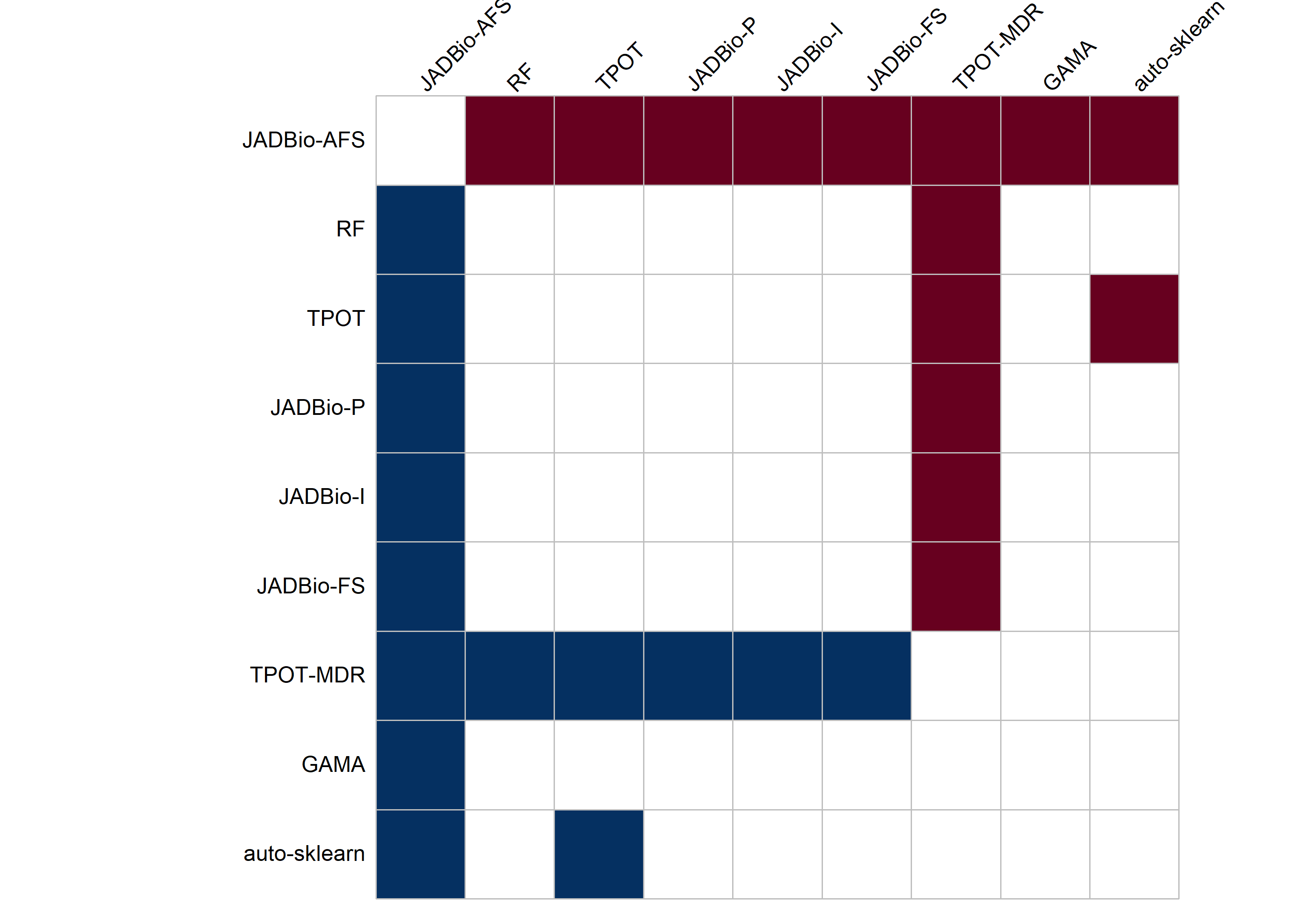


Supplementary Figure 5: statistical significance of the median difference between different tools, as assessed by Bonferroni adjusted, paired Wilcoxon signed-rank test. Blue squares indicates that the tool on the row has higher AUC (better performance) than the tool on the column, vice versa for red squares (adjusted p-value <= 0.05)


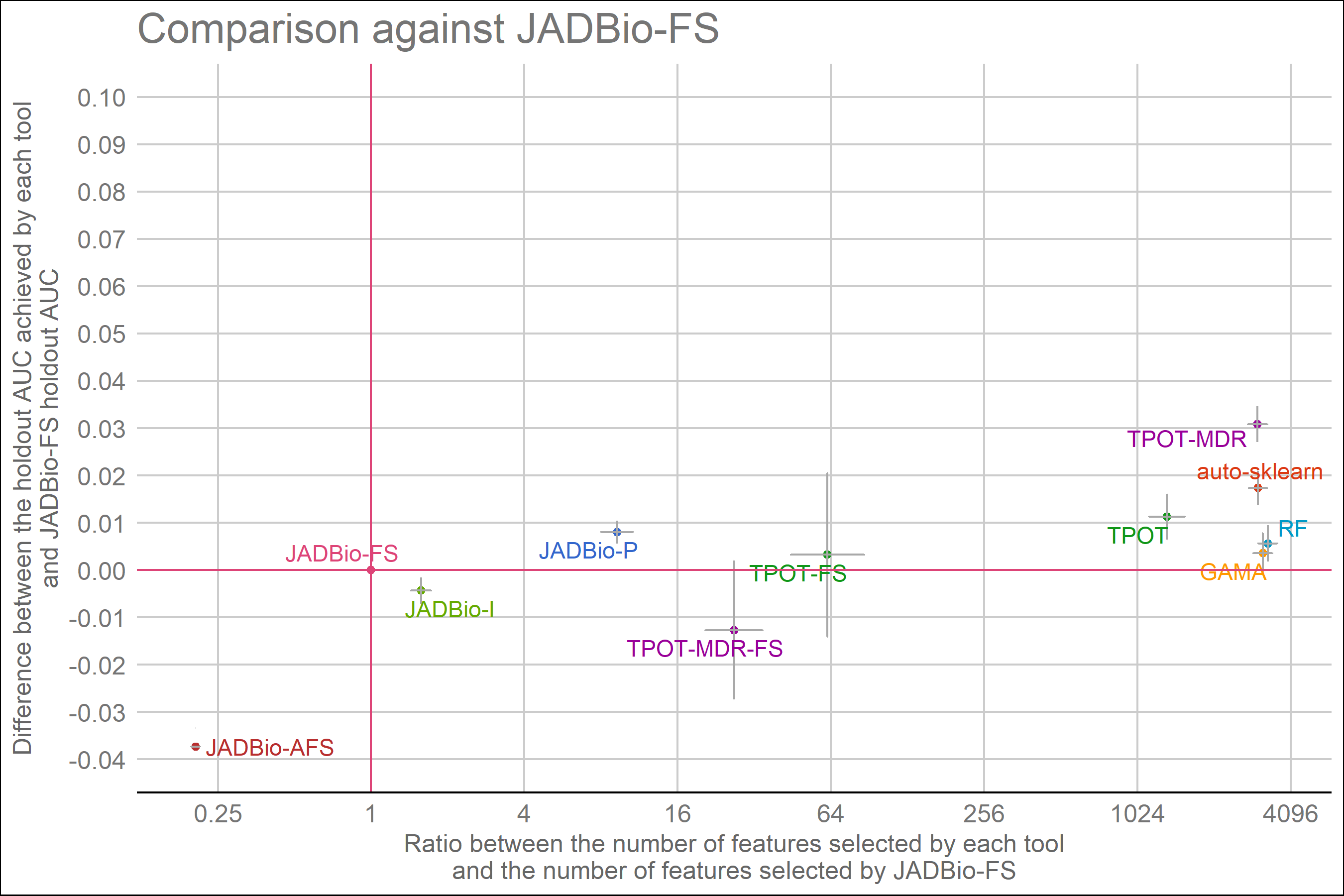


Supplementary Figure 6: Comparison between JADBio-FS and all other tools in terms of average dimensionality reduction and average performance gain. The dimensionality reduction with respect to JADBio-FS (x-axis) is computed as the ratio between the number of features selected by each tool and the number of features selected by JADBio-FS (log2 scale). Values larger than one indicate that the tool selects more features than JADBio. The performance gain is reported on the y-axis, and it is computed as the difference between the holdout AUC achieved by each tool and the holdout AUC achieved by JADBio-FS. Values larger than zero indicates that the tool performs better than JADBio-FS. Each point corresponds to a specific tool, and its coordinates are the average dimensionality reduction and the average performance gain across all runs that the specific tool has in common with JADBio-FS. Error bars are computed as average +/- standard error. Cranberry colored lines denote baseline value, i.e., relative dimensionality reduction equal to one and performance gain equal to zero


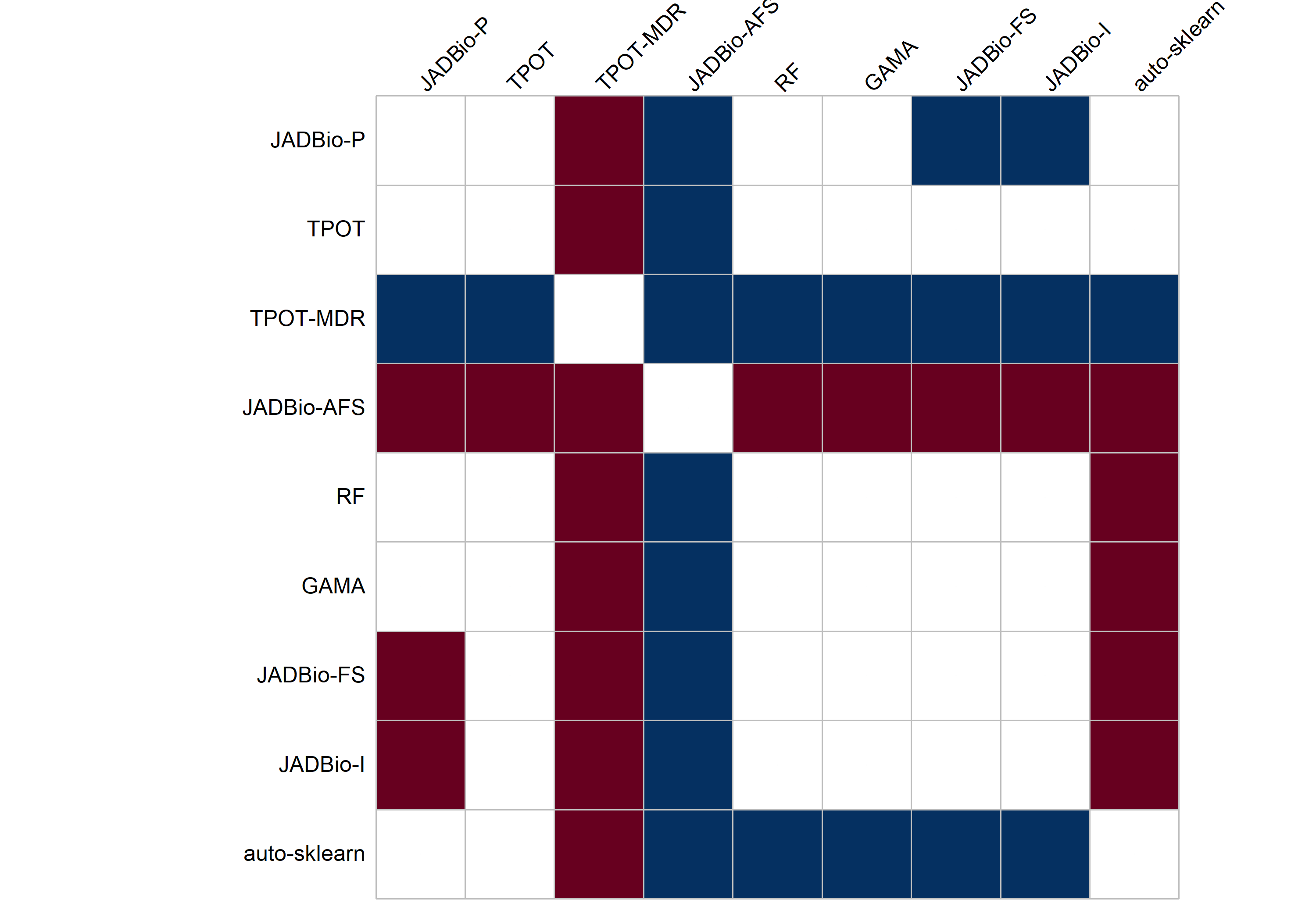


Supplementary Figure 7: statistical significance of the average difference between different tools, as assessed by Bonferroni adjusted, paired Wilcoxon signed-rank test. Blue squares indicates that the tool on the row has higher AUC (better performance) than the tool on the column, vice versa for red squares (adjusted p-value <= 0.05)


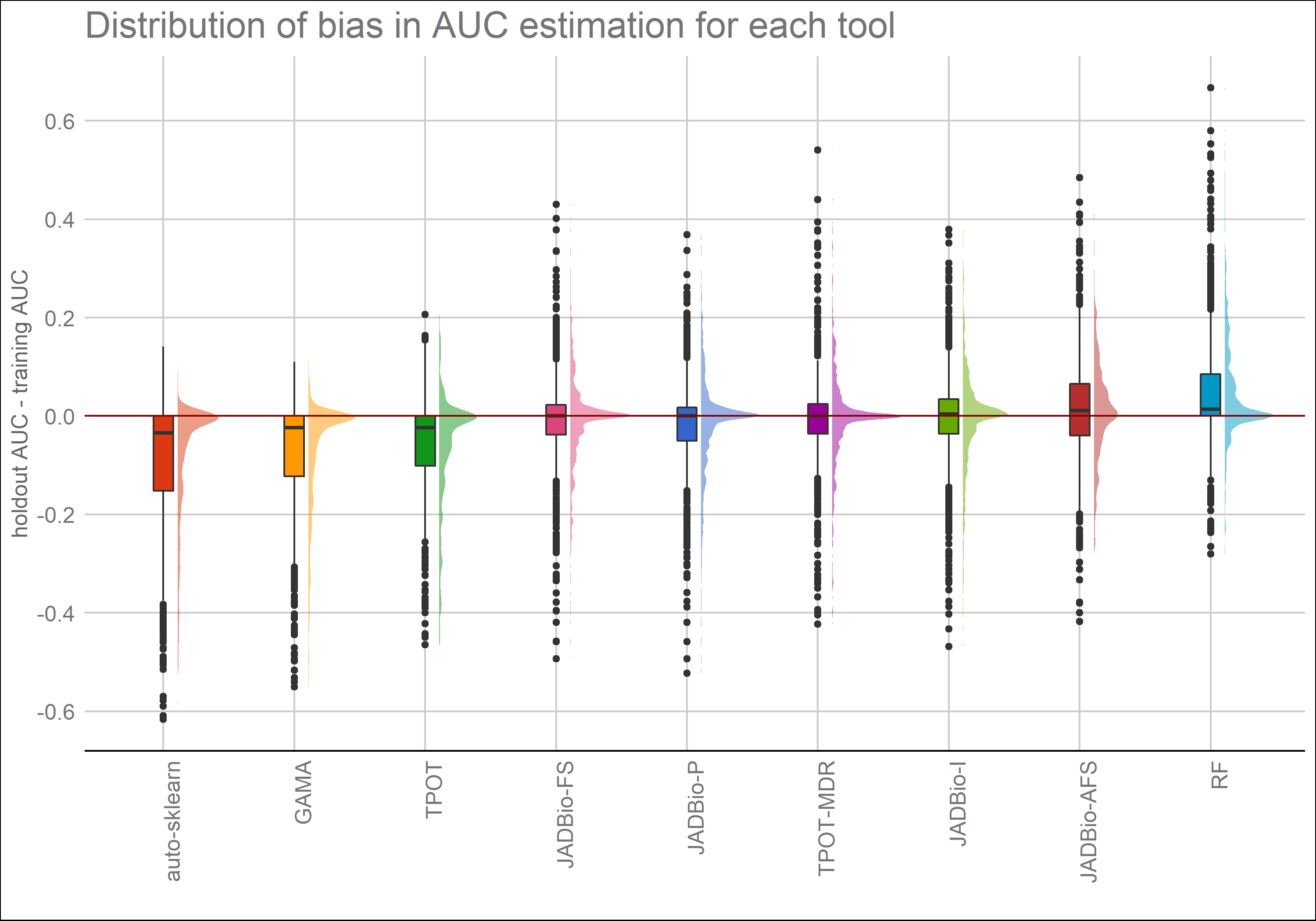


Supplementary Figure 8: AUC bias distribution across 720 classification tasks (2 tasks for each of the 360 datasets). The distribution for each tool is reported both as a box and violin plot. Bias is defined as holdout AUC minus the AUC estimated on the training set; consequently, distributions over the zero line correspond to overconservative estimates, while results below the line are overoptimistic. For each tool we selected the available estimation method that should provide the most unbiased results: BBC for JADBio, Out Of Bag (OOB) for Random Forest, holdout set for auto-sklearn and CV for all others.

# Supplementary Tables

Supplementary Table 1: contrasting JADBio and Wirbel et al^1^ results. The upper portion of the table reports the predictive performances obtained by JADBio; each cell contains the AUC value obtained by applying the model trained on the row cohort on the column cohort. Values on the main diagonal (in blue) are estimated on the training set through the BBC algorithm^2^. The rightmost column reports the mean AUC computed on the validation sets. The middle portion of the tables reports the predictive performances obtained by Wirbel et al; results are organized as in the upper portion, with training estimates obtained through nested cross validation. The bottom portion reports the cell-to-cell difference between the two approaches, with positive values indicating JADBio having better results.

| JADBio | FR | AT | CN | US | DE | Mean validation |
| --- | --- | --- | --- | --- | --- | --- |
| FR | 0.845 | 0.75 | 0.776 | 0.698 | 0.755 | 0.745 |
| AT | 0.66 | 0.914 | 0.768 | 0.589 | 0.718 | 0.684 |
| CN | 0.771 | 0.708 | 0.845 | 0.682 | 0.834 | 0.749 |
| US | 0.751 | 0.707 | 0.733 | 0.697 | 0.813 | 0.751 |
| DE | 0.783 | 0.745 | 0.89 | 0.698 | 0.855 | 0.779 |
| Wirbel et al | *FR* | *AT* | *CN* | *US* | *DE* | *Mean* *validation* |
| FR | *0.85* | 0.76 | 0.82 | 0.64 | 0.83 | 0.7625 |
| AT | 0.62 | *0.92* | 0.74 | 0.59 | 0.65 | 0.65 |
| CN | 0.82 | 0.76 | *0.81* | 0.67 | 0.83 | 0.77 |
| US | 0.76 | 0.78 | 0.7 | *0.74* | 0.79 | 0.7575 |
| DE | 0.84 | 0.79 | 0.88 | 0.74 | *0.79* | 0.8125 |
| Difference between JADBio and Wirbel et al | *FR* | *AT* | *CN* | *US* | *DE* | *Mean* *validation* |
| FR | -0.01 | -0.01 | -0.04 | 0.058 | -0.08 | -0.018 |
| AT | 0.04 | -0.01 | 0.028 | 0 | 0.068 | 0.034 |
| CN | -0.05 | -0.05 | 0.035 | 0.012 | 0.004 | -0.021 |
| US | -0.01 | -0.07 | 0.033 | -0.04 | 0.023 | -0.006 |
| DE | -0.06 | -0.05 | 0.01 | -0.04 | 0.065 | -0.033 |
| Mean validation | -0.02 | -0.045 | 0.008 | 0.007 | 0.004 | -0.009 |

Supplementary Table 2: links to JADBio results for the colorectal cancer microbiome use case. Each link points to an interactive web page where the results of the respective cohort can be explored.

| Cohort | Link |
| --- | --- |
| AT | <https://app.jadbio.com/share/4ce00fe4-9a2c-4410-8fa3-2ffcd7655ed5> |
| CN | <https://app.jadbio.com/share/f138ced3-1357-465b-81f0-523e64a3abf7> |
| DE | <https://app.jadbio.com/share/1d4f56db-51f2-4d6f-934d-c254c8c4b0cc> |
| FR | <https://app.jadbio.com/share/e9f1e753-2656-4d3c-8f60-8af7427906f7> |
| US | <https://app.jadbio.com/share/5fe41835-b69b-4ede-8e54-f6bba9c167bf> |

Supplementary Table 3: number of successful runs for each AutoML tool, out of 720 analyses. The number of runs failed because of internal errors or reached time limit is reported as well.

| Tool | Number of successful runs | Number of runs failed for internal error | Number of runs failed for time limit |
| --- | --- | --- | --- |
| TPOT-MDR | 645 (89.6%) | 54 (7.5%) | 21 (2.9%) |
| auto-sklearn | 667 (92.6%) | 24 (3.3%) | 29 (4%) |
| JADBio-P | 720 (100%) | 0 (0%) | 0 (0%) |
| GAMA | 696 (96.7%) | 10 (1.4%) | 14 (1.9%) |
| Random Forest | 720 (100%) | 0 (0%) | 0 (0%) |
| JADBio-FS | 720 (100%) | 0 (0%) | 0 (0%) |
| JADBio-I | 720 (100%) | 0 (0%) | 0 (0%) |
| TPOT | 318 (44.2%) | 303 (42%) | 99 (13.8%) |
| JADBio-AFS | 720 (100%) | 0 (0%) | 0 (0%) |
| AutoPrognosis | 0 (0%) | 572 (79.4%) | 148 (20.6%) |
| Total | 5926 | 963 | 311 |

Supplementary Table 4: bootstrapped distribution of the median difference in holdout AUC between each pair of tools, along with statistical significance of the difference between tools in terms of holdout AUC. Each row reports the comparison between two different tools; for each comparison we report the median difference in holdout AUC, along with its 95% confidence interval, a p-value quantifying the statistical significance of the median difference, and the corresponding Bonferroni adjusted p-value. Median AUC is computed in a pairwise way, i.e., by first subtracting the holdout AUC values across common successful runs and then computing the median of the resulting vector. The name of each row indicates the order of the subtraction, i.e., Tool1 – Tool2. The p-value is computed through a permutation approach, details in the Supplementary Methods.

| Tools (Tool 1 – Tool 2) | Median AUC difference | 2.5% Quantile | 97.5%  Quantile | p-value | Adjusted p-value |
| --- | --- | --- | --- | --- | --- |
| TPOT-MDR - auto-sklearn | 0 | 0 | 0 | 1 | 1 |
| TPOT-MDR - JADBio-P | 0.001245 | 0 | 0.004529 | 0 | 0 |
| TPOT-MDR - GAMA | 0 | 0 | 1.16E-06 | 1 | 1 |
| TPOT-MDR – RF | 0.004058 | 0.000494 | 0.006768 | 0 | 0 |
| TPOT-MDR - JADBio-FS | 0.003361 | 0.000352 | 0.008518 | 0 | 0 |
| TPOT-MDR - JADBio-I | 0.005682 | 0.001421 | 0.00889 | 0 | 0 |
| TPOT-MDR – TPOT | 0.001865 | 0 | 0.006543 | 1.00E-04 | 0.0036 |
| TPOT-MDR - JADBio-AFS | 0.032743 | 0.027549 | 0.04142 | 0 | 0 |
| auto-sklearn - JADBio-P | 0 | 0 | 0.000193 | 1 | 1 |
| auto-sklearn – GAMA | 0 | 0 | 0 | 1 | 1 |
| auto-sklearn – RF | 0 | 0 | 0.002551 | 1 | 1 |
| auto-sklearn - JADBio-FS | 0 | 0 | 0.00103 | 1 | 1 |
| auto-sklearn - JADBio-I | 0 | 0 | 0.001894 | 1 | 1 |
| auto-sklearn - TPOT | 0.002191 | 0 | 0.004585 | 8.00E-04 | 0.0288 |
| auto-sklearn - JADBio-AFS | 0.029167 | 0.02381 | 0.034413 | 0 | 0 |
| JADBio-P – GAMA | 0 | 0 | 0 | 1 | 1 |
| JADBio-P – RF | 0 | 0 | 0 | 1 | 1 |
| JADBio-P - JADBio-FS | 0 | 0 | 0 | 1 | 1 |
| JADBio-P - JADBio-I | 0 | 0 | 0 | 1 | 1 |
| JADBio-P – TPOT | 0 | -0.00015 | 0 | 1 | 1 |
| JADBio-P - JADBio-AFS | 0.014044 | 0.009782 | 0.020953 | 0 | 0 |
| GAMA - RF | 0 | 0 | 0 | 1 | 1 |
| GAMA - JADBio-FS | 0 | 0 | 0 | 1 | 1 |
| GAMA - JADBio-I | 0 | 0 | 0.000885 | 1 | 1 |
| GAMA - TPOT | 0 | 0 | 3.33E-06 | 1 | 1 |
| GAMA - JADBio-AFS | 0.021567 | 0.016548 | 0.028576 | 0 | 0 |
| RF - JADBio-FS | 0 | 0 | 0 | 1 | 1 |
| RF - JADBio-I | 0 | 0 | 0 | 1 | 1 |
| RF - TPOT | 0 | -0.00065 | 0.001892 | 1 | 1 |
| RF - JADBio-AFS | 0.021209 | 0.015229 | 0.026917 | 0 | 0 |
| JADBio-FS - JADBio-I | 0 | 0 | 0 | 1 | 1 |
| JADBio-FS - TPOT | 0 | -0.00132 | 0 | 1 | 1 |
| JADBio-FS - JADBio-AFS | 0.010561 | 0.007342 | 0.015119 | 0 | 0 |
| JADBio-I - TPOT | -0.0024 | -0.00694 | 0 | 0.0027 | 0.0972 |
| JADBio-I - JADBio-AFS | 0.012896 | 0.008008 | 0.018666 | 0 | 0 |
| TPOT - JADBio-AFS | 0.016484 | 0.00905 | 0.026499 | 0 | 0 |

Supplementary Table 5: bootstrapped distribution of the average difference in holdout AUC between each pair of tools, along with statistical significance of the difference between tools in terms of holdout AUC. Each row reports the comparison between two different tools; for each comparison we report the average difference in holdout AUC, along with its 95% confidence interval, a p-value quantifying the statistical significance of the average difference, and the corresponding Bonferroni adjusted p-value. Average AUC is computed in a pairwise way, i.e., by first subtracting the holdout AUC values across common successful runs and then computing the mean of the resulting vector. The name of each row indicates the order of the subtraction, i.e., Tool1 – Tool2. The p-value is computed through a permutation approach, details in the Supplementary Methods.

| Tools (Tool 1 – Tool 2) | Average AUC difference | 2.5% Quantile | 97.5%  Quantile | p-value | Adjusted p-value |
| --- | --- | --- | --- | --- | --- |
| TPOT-MDR - auto-sklearn | 0.011409 | 0.005878 | 0.017119 | 1.00E-04 | 0.0036 |
| TPOT-MDR - JADBio-P | 0.022647 | 0.015137 | 0.029839 | 0 | 0 |
| TPOT-MDR - GAMA | 0.020161 | 0.013022 | 0.027126 | 0 | 0 |
| TPOT-MDR – RF | 0.024165 | 0.017563 | 0.030412 | 0 | 0 |
| TPOT-MDR - JADBio-FS | 0.03133 | 0.023439 | 0.038653 | 0 | 0 |
| TPOT-MDR - JADBio-I | 0.03517 | 0.026977 | 0.04257 | 0 | 0 |
| TPOT-MDR – TPOT | 0.016024 | 0.007016 | 0.02454 | 4.00E-04 | 0.0144 |
| TPOT-MDR - JADBio-AFS | 0.068969 | 0.059794 | 0.078228 | 0 | 0 |
| auto-sklearn - JADBio-P | 0.009529 | 0.002941 | 0.015428 | 0.0022 | 0.0792 |
| auto-sklearn – GAMA | 0.01648 | 0.008811 | 0.024366 | 0 | 0 |
| auto-sklearn – RF | 0.012768 | 0.006528 | 0.018432 | 1.00E-04 | 0.0036 |
| auto-sklearn - JADBio-FS | 0.018441 | 0.010979 | 0.025167 | 0 | 0 |
| auto-sklearn - JADBio-I | 0.022134 | 0.015263 | 0.028727 | 0 | 0 |
| auto-sklearn - TPOT | 0.012575 | 0.003234 | 0.022359 | 0.0097 | 0.3492 |
| auto-sklearn - JADBio-AFS | 0.058344 | 0.049883 | 0.067357 | 0 | 0 |
| JADBio-P – GAMA | 0.004321 | -0.00315 | 0.010928 | 0.2649 | 1 |
| JADBio-P – RF | 0.001813 | -0.00471 | 0.00897 | 0.6081 | 1 |
| JADBio-P - JADBio-FS | 0.008647 | 0.003864 | 0.013702 | 1.00E-04 | 0.0036 |
| JADBio-P - JADBio-I | 0.011856 | 0.006061 | 0.017876 | 2.00E-04 | 0.0072 |
| JADBio-P – TPOT | -0.00212 | -0.01049 | 0.005812 | 0.62 | 1 |
| JADBio-P - JADBio-AFS | 0.046906 | 0.039257 | 0.054703 | 0 | 0 |
| GAMA - RF | -0.00313 | -0.01101 | 0.004469 | 0.4294 | 1 |
| GAMA - JADBio-FS | 0.004547 | -0.00333 | 0.012871 | 0.294 | 1 |
| GAMA - JADBio-I | 0.008026 | -0.00012 | 0.016289 | 0.0546 | 1 |
| GAMA - TPOT | -0.00081 | -0.01006 | 0.007317 | 0.8585 | 1 |
| GAMA - JADBio-AFS | 0.04245 | 0.033727 | 0.052409 | 0 | 0 |
| RF - JADBio-FS | 0.006834 | -0.00094 | 0.014361 | 0.0789 | 1 |
| RF - JADBio-I | 0.010044 | 0.001912 | 0.017736 | 0.0115 | 0.414 |
| RF - TPOT | 0.000277 | -0.00988 | 0.010732 | 0.9575 | 1 |
| RF - JADBio-AFS | 0.045094 | 0.036139 | 0.054142 | 0 | 0 |
| JADBio-FS - JADBio-I | 0.003209 | -0.00228 | 0.008342 | 0.2733 | 1 |
| JADBio-FS - TPOT | -0.01284 | -0.02319 | -0.00353 | 0.0085 | 0.306 |
| JADBio-FS - JADBio-AFS | 0.038259 | 0.030938 | 0.045773 | 0 | 0 |
| JADBio-I - TPOT | -0.00906 | -0.01738 | -0.00048 | 0.0416 | 1 |
| JADBio-I - JADBio-AFS | 0.03505 | 0.027301 | 0.042678 | 0 | 0 |
| TPOT - JADBio-AFS | 0.042814 | 0.030932 | 0.055163 | 0 | 0 |

Supplementary Table 6: bootstrapped distribution of the median estimation bias for each tool. Bias is defined as the difference between the AUC achieved on the holdout set and the performance estimate computed on the training set. Each row reports the median AUC bias, along with its 95% confidence interval, a p-value assessing whether the median bias is different from zero in as statistically significant way, and the corresponding Bonferroni adjusted p-value. The p-value is computed through a permutation approach, details in the Supplementary Methods.

| Tool | Median bias | 2.5% Quantile | 97.5% Quantile | p-value | Adjusted p-value |
| --- | --- | --- | --- | --- | --- |
| GAMA | -0.024 | -0.03361 | -0.01713 | 0 | 0 |
| auto-sklearn | -0.03478 | -0.04563 | -0.02222 | 0 | 0 |
| TPOT | -0.02387 | -0.04 | -0.01267 | 0 | 0 |
| JADBio-P | 0 | 0 | 0 | 1 | 1 |
| JADBio-I | 0.003727 | 0.001422 | 0.005908 | 0.0012 | 0.0108 |
| JADBio-FS | 0 | 0 | 0.001026 | 1 | 1 |
| JADBio-AFS | 0.010766 | 0.005161 | 0.015509 | 0 | 0 |
| TPOT-MDR | 0 | 0 | 0 | 1 | 1 |
| RF | 0.01378 | 0.009091 | 0.022679 | 0 | 0 |

Supplementary Table 7: bootstrapped distribution of the average estimation bias for each tool. Bias is defined as the difference between the AUC achieved on the holdout set and the performance estimate computed on the training set. Each row reports the average AUC bias, along with its 95% confidence interval, a p-value assessing whether the average bias is different from zero in as statistically significant way, and the corresponding Bonferroni adjusted p-value. The p-value is computed through a permutation approach, details in the Supplementary Methods.

| Tool | Average bias | 2.5% Quantile | 97.5% Quantile | p-value | Adjusted p-value |
| --- | --- | --- | --- | --- | --- |
| GAMA | -0.07935 | -0.08884 | -0.07069 | 0 | 0 |
| auto-sklearn | -0.09727 | -0.10785 | -0.08698 | 0 | 0 |
| TPOT | -0.06511 | -0.07837 | -0.05317 | 0 | 0 |
| JADBio-P | -0.01759 | -0.02526 | -0.01046 | 0 | 0 |
| JADBio-I | -0.00518 | -0.01257 | 0.002663 | 0.1804 | 1 |
| JADBio-FS | -0.00862 | -0.0162 | -0.00144 | 0.029 | 0.261 |
| JADBio-AFS | 0.012822 | 0.004256 | 0.020757 | 0.0018 | 0.0162 |
| TPOT-MDR | -0.00365 | -0.01146 | 0.004799 | 0.3784 | 1 |
| RF | 0.050853 | 0.042768 | 0.059102 | 0 | 0 |

Supplementary Table 8: bootstrapped distribution of the median estimation bias for each tool, computed on 196 “difficult” tasks, i.e., tasks where RF achieved less than 0.8 AUC on the holdout set. Bias is defined as the difference between the AUC achieved on the holdout set and the performance estimate computed on the training set. Each row reports the median AUC bias, along with its 95% confidence interval, a p-value assessing whether the median bias is different from zero in as statistically significant way, and the corresponding Bonferroni adjusted p-value. The p-value is computed through a permutation approach, details in the Supplementary Methods.

| Tool | Median bias | 2.5% Quantile | 97.5% Quantile | p-value | Adjusted p-value |
| --- | --- | --- | --- | --- | --- |
| GAMA | -0.17797 | -0.20814 | -0.15253 | 0 | 0 |
| auto-sklearn | -0.24956 | -0.28946 | -0.21017 | 0 | 0 |
| TPOT | -0.12957 | -0.16111 | -0.09236 | 0 | 0 |
| JADBio-P | -0.05695 | -0.07504 | -0.03743 | 1.00E-04 | 9.00E-04 |
| JADBio-I | -0.02244 | -0.04309 | 0.000888 | 0.0125 | 0.1125 |
| JADBio-FS | -0.03254 | -0.04917 | -0.00882 | 0.0074 | 0.0666 |
| JADBio-AFS | -0.00932 | -0.03037 | 0.008311 | 0.2496 | 1 |
| TPOT-MDR | -0.03644 | -0.06263 | -0.01722 | 0.0041 | 0.0369 |
| RF | 0.043528 | 0.025613 | 0.067222 | 0 | 0 |

Supplementary Table 9: maximum number of configurations for each JADBio setting.

| JADBio setting | Maximum number of configurations | |
| --- | --- | --- |
| JADBio-P | 3249 |  |
| JADBio-FS | 3017 |  |
| JADBio-AFS | 1393 |  |
| JADBio-I | 309 |  |

# Supplementary Data

Supplementary Data 1: characteristics of the datasets used for quantitative comparison across AutoML tools. For each dataset we report its identifier, the type of data (transcriptomics microarray, transcriptomics rna-seq, methylation and metabolomics), the number of features and samples, the repository from which the dataset originated from, the disease the data refer to, and the disease classification.

The table is provided as supplementary data.

# References

1. Wirbel, J. *et al.* Meta-analysis of fecal metagenomes reveals global microbial signatures that are specific for colorectal cancer. *Nature Medicine* **25**, 679–689 (2019).

2. Tsamardinos, I., Greasidou, E. & Borboudakis, G. Bootstrapping the out-of-sample predictions for efficient and accurate cross-validation. *Machine Learning* **107**, 1895–1922 (2018).
